# Ulam-Hyers stability of tuberculosis and COVID-19 co-infection model under Atangana-Baleanu fractal-fractional operator

**DOI:** 10.1038/s41598-023-35624-4

**Published:** 2023-06-02

**Authors:** Arunachalam Selvam, Sriramulu Sabarinathan, Beri Venkatachalapathy Senthil Kumar, Haewon Byeon, Kamel Guedri, Sayed M. Eldin, Muhammad Ijaz Khan, Vediyappan Govindan

**Affiliations:** 1 Department of Mathematics, Faculty of Engineering and Technology, SRM Institute of Science and Technology, Kattankulthur, Tamilnadu 603 203 India; 2 Department of Mathematics, Saveetha School of Engineering, Saveetha Institute of Medical and Technical Sciences, Chennai, Tamilnadu 602 105 India; 3 Department of Digital Anti-Aging Healthcare (BK21), Inje University, Gimhae, 50834 Republic of Korea; 4 Mechanical Engineering Department, College of Engineering and Islamic Architecture, Umm Al-Qura University, P.O. Box 5555, Makkah, 21955 Saudi Arabia; 5 Center of Research, Faculty of Engineering, Future University in Egypt, New Cairo, 11835 Egypt; 6 Laboratry of Systems Ecology and Sustainability Science College of Engineering, Peking University, Beijing, China; 7 Department of Mathematics, DMI St John The Baptist University Central Mangochi-409, Cental Africa, Malawi

**Keywords:** Mathematics and computing, Applied mathematics

## Abstract

The intention of this work is to study a mathematical model for fractal-fractional tuberculosis and COVID-19 co-infection under the Atangana-Baleanu fractal-fractional operator. Firstly, we formulate the tuberculosis and COVID-19 co-infection model by considering the tuberculosis recovery individuals, the COVID-19 recovery individuals, and both disease recovery compartment in the proposed model. The fixed point approach is utilized to explore the existence and uniqueness of the solution in the suggested model. The stability analysis related to solve the Ulam-Hyers stability is also investigated. This paper is based on Lagrange’s interpolation polynomial in the numerical scheme, which is validated through a specific case with a comparative numerical analysis for different values of the fractional and fractal orders.

## Introduction

SARS-CoV-2 (COVID-19) erupted in Wuhan City, China, in late 2019 and evolved into a global pandemic. More than 220 countries and territories worldwide are affected by the COVID-19 pandemic, which affects every part of our daily lives. In the 21st century, human COVIDs like SARS-CoV and MERS-CoV have risen from the creature supply-induced worldwide pandemic with an alarming death rate and morbidity. The quantities of contaminated cases passing despite everything increment essentially and do not indicate a very controlled circumstance as of 25th October 2022, a total aggregate of 62,753,838 (65,782,318) contaminated (deceased) COVID-19 cases were accounted for all over the world. These are essentially partitioned into four genera: $\alpha , \beta , \gamma ,$ and $\delta$. If $\alpha$ is $\beta$-CoV for the most part, tainted vertebrates, during $\gamma$ is $\delta$-CoV turned to influence birds. Furthermore, HCoV-229E and HCoV-NL63 of $\alpha$-CoVs and HCoV-HKU1, and HCoV-OC43 of $\beta$-CoVs, exhibit low pathogenicity as well as moderate respiratory side effects as typical viruses. The other two recognizable $\beta$-CoVs, like MERS-CoV and SARS-CoV display intense and dangerous respiratory illnesses^1^.

Tuberculosis (shortly TB) is one of the most deadly diseases. The physiology of Mycobacterium tuberculosis is the causative agent of this life-threatening disease. However, it may harm glands, bones, the brain, the kidneys and other organs. Mycobacterium tuberculosis flourished through various stages and its host then was East Africa. Early TB infection originated in East Africa 3 million years ago and it was concluded that it might have spread to early primates around then. The incidence of TB reportedly dates back over 5000 years. Globally, 1.45 million people died and more than 10 million became infected with tubercular bacilli, which made it the world’s leading infectious killer in 2018^2^.

Ulam^3^, in his celebrated talk in 1940 in the mathematics club of the University of Wisconsin, presented a number of uncertain issues. The following year, Hyers^4^ was the first mathematician to answer Ulam’s question concerning the stability of functional equations. Along these lines, Rassias^5^ autonomously presented the generalization of the Hyers theorem contained in the unbounded Cauchy contrast in 1978. Following this outcome, many mathematicians have investigated the expansion of the Ulam stability with other functional and differential equations using various techniques in different directions (see also^6–9^).

The bifurcation of fractional order has also been related to practical ventures. It is extensively employed in 4*D* neural network incorporating two different time delays^10^, three triangles multi-delayed neural network^11^ and delayed BAM neural network^12^. Authors in^13^ proposed the interaction between the immune system and cancer cells. The tumor-immune model has been investigated from a numerical and theoretical point of view. A fractal-fractional model of tumor-immune interaction was discussed in^14^.

Goudiaby et al.^15^ observed the simple mathematical model of COVID-19 and tuberculosis co-infection with treatment for the infected. They incorporated the optimal control system into a sub-model using five control compartments. Dokuyucu and Dutta^16^ analyzed a model of fractional derivative type Ebola virus spread that leads to disease in Africa by using the Caputo-Fabrizio operator. They examined the numerical solutions for the proposed model by using the Adam-Basford method for the Caputo-Fabrizio fractional derivative operator.

Mekonen et al.^17^ examined the COVID-19 and tuberculosis co-dynamics model and a numerical simulation showed the effect of various values of fractional order and compared the sensitive parameters. In^18^, the authors analyzed the COVID-19 and tuberculosis co-infection of optimal control problems. Zhang et al.^19^ investigated the Caputo derivative fractal-fractional type, anthropogenic cutaneous leishmania model. Based on fractional derivative order, they analyzed the existence, uniqueness and Hyers-Ulam stability of the solution derived for the model (see also^20–23^).

Aziz Khan et al.^24^ studied that COVID-19 disease spreads from person to person with the help of the nabla Atangana-Baleanu-Caputo fractional derivative of Ulam-Hyers stability and optimal control strategies. In recent years, many researchers have studied the fractional model of Ulam stability with fractional results and related papers (see also^25–30^).

Amin et al.^31^ examined a fractal-fractional type COVID-19 model under the Atangana-Baleanue fractal-fractional operator. Then, they analyzed the existence, uniqueness and Ulam-Hyers stability of the solution derived for the model with various values of $k_1$ and $k_2$. Asamoah et al.^32^ provided the existence and uniqueness of the solutions and Ulam-Hyers stability using the fractal-fractional Atangana-Baleanu derivative for the *Q* fever disease of complex dynamics.

Hasib Khan et al.^33^ provided a fractal-fractional order TB model restricted to a case study in China. The authors derived the Ulam-Hyers stability of advanced fractal-fractional operators. Then, they used the Lagrange polynomials interpolation numerical scheme based on the obtained algorithms. In^34^, the authors observed the HIV-TB co-infection model using the fractional order of the Atangana-Baleanu derivative.

The objective of this study is to utilize our numerical algorithm to observe the impact of two different orders on the approximate solutions of the given model. These two orders, namely the fractal dimension and fractional order are critical components of mathematical models that use fractional orders for simulation. This study marks the initial investigation of fractal-fractional TB and COVID-19 co-infection model using advanced fractal-fractional operators, explicitly focusing on Ulam-Hyers stability. The model also provides the existence, uniqueness, and Ulam-Hyers stability of the solutions in the proposed model. Our findings from various fractional mathematical models have motivated us to enhance our numerical approaches to accommodate fractal-fractional simulations.

## Basic definitions

In this segment, we will discuss some basic concepts related to the fractal-fractional operator and some known definitions that will be needed to obtain the main results of this study. Also, in this work, we assume the space $\{y(s)\in \mathbb {C} ([0,1])\rightarrow \mathbb {R}\}$ with $\Vert y\Vert =max_{s\in [0,1]}|y(s)|$.

### Definition 1

Let $y\in \mathbb {C}((a,b),\mathbb {R})$ be a fractal differentiable on (*a*, *b*). The fractal-fractional derivative of *y*(*s*) with fractional order $0<k_1\le 1$ and fractal dimension $0<k_2\le 1$ in the sense of Atangana-Baleanu having a generalized Mittag-Leffler type kernel can be defined as follows^33^:

$$ {}^{{\mathscr {F}}{\mathscr {F}}{\mathscr {M}}}_{\,\,\,\,\,\,\,\,\,\,\,\,\text {0}}\mathscr {D}_{s}^{k_1, k_2}\Big (y(s)\Big )=\frac{{\mathscr {A}}{\mathscr {B}}(k_1)}{(1-k_1)}\frac{d}{ds^{k_2}}\int _{0}^{s}y(u)\mathscr {E}_{k_1}\left( \frac{-k_1}{1-k_1}\left( s-u\right) ^{k_1}\right) du, $$

where $$\mathscr{A}\mathscr{B}(k_1)=1-k_1+\frac{k_1}{\Gamma (k_1)}$$ and $$\frac{dy(u)}{du^{k_2}}=\lim _{s\rightarrow u} \frac{y(s)-y(u)}{s^{k_2}-u^{k_2}}$$.

### Definition 2

For the same function *y*, considered above, the fractal-fractional integral of *y*(*s*) with fractional order $0<k_1\le 1$ in the sense of Atangana-Baleanu having a Mittag-Leffler type kernel can be defined as follows^31^:

$$\begin{aligned} {}^{{\mathscr {F}}{\mathscr {F}}{\mathscr {M}}}_{\,\,\,\,\,\,\,\,\,\,\,\,\text {0}}\mathscr {I}_{s}^{k_1, k_2}\Big (y(s)\Big )=\frac{k_1k_2}{{\mathscr {A}}{\mathscr {B}}(k_1)\Gamma (k_1)}\int _{0}^{s} u^{k_2-1} y(u)(s-u)^{k_1-1}\ du+\frac{k_2(1-k_1)s^{k_2-1}}{\mathscr{A}\mathscr{B}(k_1)}y(s). \end{aligned}$$

### Ethical approval

This article does not contain any studies with human participants or animals performed by any of the authors.

## Model formulation

This segment describes a TB and COVID-19 co-infection model based on the Atangana-Baleanu fractal-fractional operator. Our model given below is an extension of some specified in^17,18^ by,

- Including the COVID-19 disease reinfection of recovered individuals; and
- Including the TB recovery compartment, the COVID-19 recovery compartment and both diseases recovery compartments; and
- Including the COVID-19 infection after recovery from TB and TB infection after recovery from COVID-19.

**Figure 1:**
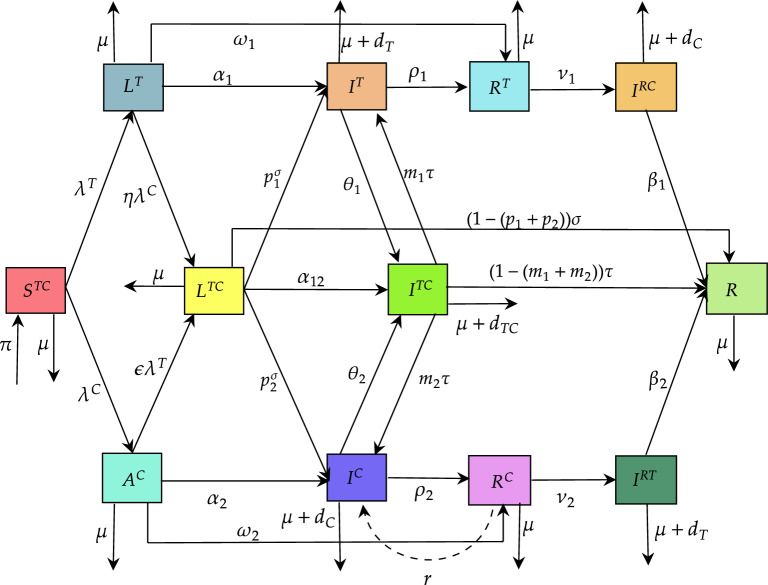
TB and COVID-19 co-infection model showing the compartments.

Under the schematic diagram given in Fig. 1, the TB and COVID-19 co-infection model is presented by the system of equations depicted as follows:

1 $$\begin{aligned} {\left\{ \begin{array}{ll} {}^{{\mathscr {F}}{\mathscr {F}}{\mathscr {M}}}_{\,\,\,\,\,\,\,\,\,\,\,\,\text {0}}{\mathscr {D}}_{s}^{k_1,k_2}S^{TC}(s)&{}=\pi -\big (\lambda ^{T}+\lambda ^{C}+\mu \big )S^{TC},\\ {}^{{\mathscr {F}}{\mathscr {F}}{\mathscr {M}}}_{\,\,\,\,\,\,\,\,\,\,\,\,\text {0}}{\mathscr {D}}_{s}^{k_1,k_2}L^{T}(s)&{}=\lambda ^{T} S^{TC}-\big (\alpha _{1}+\omega _{1}+\eta \lambda ^{C}+\mu \big ) L^{T},\\ {}^{{\mathscr {F}}{\mathscr {F}}{\mathscr {M}}}_{\,\,\,\,\,\,\,\,\,\,\,\,\text {0}}{\mathscr {D}}_{s}^{k_1,k_2}I^{T}(s)&{}=\alpha _1 L^{T}+ p_1^{\sigma } L^{TC}+m_1 \tau I^{TC}-\big (\rho _1+\theta _1+\mu +d^{T}\big )I^{T},\\ {}^{{\mathscr {F}}{\mathscr {F}}{\mathscr {M}}}_{\,\,\,\,\,\,\,\,\,\,\,\,\text {0}}{\mathscr {D}}_{s}^{k_1,k_2}R^{T}(s)&{}=\omega _1 L^{T}+\rho _1 I^{T}-\big (\nu _1+\mu \big ) R^{T},\\ {}^{{\mathscr {F}}{\mathscr {F}}{\mathscr {M}}}_{\,\,\,\,\,\,\,\,\,\,\,\,\text {0}}{\mathscr {D}}_{s}^{k_1,k_2}I^{RC}(s)&{}=\nu _1 R^{T}-\big (\beta _1+\mu +d^C\big ) I^{RC},\\ {}^{{\mathscr {F}}{\mathscr {F}}{\mathscr {M}}}_{\,\,\,\,\,\,\,\,\,\,\,\,\text {0}}{\mathscr {D}}_{s}^{k_1,k_2}A^{C}(s)&{}=\lambda ^C S^{TC}-\big (\alpha _2+\omega _2+\epsilon \lambda ^T +\mu \big ) A^C,\\ {}^{{\mathscr {F}}{\mathscr {F}}{\mathscr {M}}}_{\,\,\,\,\,\,\,\,\,\,\,\,\text {0}}{\mathscr {D}}_{s}^{k_1,k_2}I^{C}(s)&{}=\alpha _2 A^C+ p_2^{\sigma } L^{TC}+m_2 \tau I^{TC}+ r R^C-(\rho _2+\theta _2+\mu +d^C)I^C,\\ {}^{{\mathscr {F}}{\mathscr {F}}{\mathscr {M}}}_{\,\,\,\,\,\,\,\,\,\,\,\,\text {0}}{\mathscr {D}}_{s}^{k_1,k_2}R^{C}(s)&{}=\omega _2 A^C+\rho _2 I^C-\big (\nu _2 +r+\mu \big )R^C,\\ {}^{{\mathscr {F}}{\mathscr {F}}{\mathscr {M}}}_{\,\,\,\,\,\,\,\,\,\,\,\,\text {0}}{\mathscr {D}}_{s}^{k_1,k_2}I^{RT}(s)&{}=\nu _2 R^C-\big (\beta _2+\mu +d^T\big ) I^{RT},\\ {}^{{\mathscr {F}}{\mathscr {F}}{\mathscr {M}}}_{\,\,\,\,\,\,\,\,\,\,\,\,\text {0}}{\mathscr {D}}_{s}^{k_1,k_2}L^{TC}(s)&{}=\eta \lambda ^C L^T+\epsilon \lambda ^T A^C- \big (\alpha _{12}+ \sigma +\mu \big ) L^{TC},\\ {}^{{\mathscr {F}}{\mathscr {F}}{\mathscr {M}}}_{\,\,\,\,\,\,\,\,\,\,\,\,\text {0}}{\mathscr {D}}_{s}^{k_1,k_2}I^{TC}(s)&{}=\alpha _{12} L^{TC}+ \theta _1 I^T+ \theta _2 I^C - \big (\tau +\mu +d^{TC}\big ) I^{TC},\\ {}^{{\mathscr {F}}{\mathscr {F}}{\mathscr {M}}}_{\,\,\,\,\,\,\,\,\,\,\,\,\text {0}}{\mathscr {D}}_{s}^{k_1,k_2}R(s)&{}=\beta _1 I^{RC}+\beta _2 I^{RT}+\big (1-\big (p_1+p_2 \big )\big )\sigma L^{TC}+\big (1-\big (m_1+m_2 \big )\big )\tau I^{TC}-\mu R.\\ \end{array}\right. } \end{aligned}$$

where

$$\begin{aligned} \begin{aligned} \lambda ^T&=\frac{\lambda _1}{N(s)}\big (L^T+I^T\big ),\\ \lambda ^C&=\frac{\lambda _2}{N(s)}\big (A^C+I^C+L^{TC}+I^{TC}\big ), \end{aligned} \end{aligned}$$

and $N(s)=S^{TC}+L^T+I^T+R^T+I^{RC}+A^C+I^C+R^C+I^{RT}+L^{TC}+I^{TC}+R$. The initial condition of the TB and COVID-19 co-infection model becomes: $S^{TC}(0)= S^{TC}_0(s)$, $L^T(0) = L^T_0(s)$, $I^T(0) = I^T_0(s)$, $R^T(0)= R^T_0(s)$, $I^{RC}(0) = I^{RC}_0(s)$, $A^C(0) = A^C_0(s)$, $I^{RT}(0) = I^{RT}_0(s)$, $L^{TC}(0)=L^{TC}_0(s)$, $I^{TC}(0)=I^{TC}_0(s)$, $R(0)=R_0(s)$.

**Table 1:** The dependent parameters description of the proposed model.

Parameters	Descriptions
$\pi$	Susceptible people has recruitment rate
$\lambda _1$	Transmission rate of TB
$\lambda ^T$	Force of infection for TB
$\eta$	Latent level TB infected becoming asymptomatic infected with COVID-19
$\alpha _1$	TB infected people to become infected
$p_1$	Recovery rate for latent TB infections in $L^{TC}$
$m_1$	Recovery rate for latent TB infections in $I^{TC}$
$\theta _1$	Infection rate with COVID-19 from TB individuals
$\lambda _2$	Transmission rate of COVID-19
$\rho _1$	Rate of recovered from TB infected people
$\nu _1$	Rate of COVID-19 after recovered from TB infected people
$\beta _1$	Rate of recovered from TB and COVID-19
$\omega _1$	Recovery rate of latent TB infected people
$d^T$	Death rate due to TB infectives
$\lambda ^C$	Force of infection for COVID-19
$\epsilon$	Rate of asymptomatic infected with COVID-19 to becoming latent TB
$\alpha _2$	Asymptomatic infected people with COVID-19 to become infected
$p_2$	Recovery rate for COVID-19 infections in $L^{TC}$
$m_2$	Recovery rate for COVID-19 infections in $I^{TC}$
$\theta _2$	Infection rate with TB from COVID-19
$\rho _2$	Rate of recovery from COVID-19
$\nu _2$	Rate of TB after recovery from COVID-19
$\omega _2$	Rate of recovered from asymptomatic infected with COVID-19
*r*	Rate of COVID - 19 is reactivation
$\beta _2$	Rate of recovered from COVID-19 and TB
$d^C$	Death rate due to the COVID-19 infectives
$\alpha _{12}$	Both diseases latent infected individuals to the co-infection class
$d^{TC}$	Death rate due to the co-infection of both diseases
$\sigma$	Rate at which people leave the co-infection in $L^{TC}$
$\tau$	Rate at which people leave the co-infected in $I^{TC}$
$\mu$	Natural death rate

In model (1), the human population is divided into twelve compartments: Susceptible to both TB and COVID-19 $(S^{TC})$, latent level TB infected people $(L^T)$, active level TB infected people $(I^T)$, recovered from TB $(R^T)$, COVID-19 infection after recovery from TB $(I^{RC})$, COVID-19 infected with asymptomatic $(A^C)$, COVID-19 infected with symptomatic $(I^C)$, recovered from COVID-19 $(R^C)$, TB infection after recovery from COVID-19 $(I^{RT})$, latent TB and COVID-19 dual infected compartment $(L^{TC})$, TB and COVID-19 dual infected compartment $(I^{TC})$, recovered people from both diseases (*R*). Table 1 describes the suggested model parameters.

We assumed that the susceptible people had been recruited into the constant rate $\pi$ and the susceptible class develops TB through contact with active level TB infected patients by a force of infection $\lambda ^T$, expressed as

$$\begin{aligned} \lambda ^T=\frac{\lambda _1}{N}\big (L^T+I^T \big ). \end{aligned}$$

This expression says that $\lambda _1$ represents the transmission rate of TB infection. The latent TB infection is considered asymptomatic and does not spread the disease. Similarly, susceptible people acquire infection with COVID-19 following effective contact with people infected with COVID-19 at a force of infection for COVID-19 $\lambda ^C$, given as

$$\begin{aligned} \lambda ^C=\frac{\lambda _2}{N} \big (A^C+I^C+L^{TC}+I^{TC} \big ). \end{aligned}$$

here $\lambda _2$ denotes the COVID-19 disease transmission rate. Furthermore, we considered the individuals in the latent level TB infected people compartment ($L^T$) leave to active level TB infected people compartment ($I^T$) at a rate of latent TB infected people to become infected $\alpha _1$, and to both diseases latent infection compartment at a force of infection $\eta \lambda ^T$ and some component is the rate of recovered from latent TB infected people $\omega _1$. Additionally, individuals with the TB disease infection ($I^T$) after recovering from active TB at a rate of $\rho _1$ while the remaining component shifted to both diseases infection ($I^{TC}$) at both diseases infectious rate of $\theta _1$ or TB infected people die due to the death rate of $d^T$. The recovered from TB ($R^T$) has left either compartment ($I^{RC}$), $\nu _1$ is respectively, the rate of COVID-19 infection after recovery from TB. Then both diseases infected in latent level ($I^{RC}$) move to the compartment (*R*) at a rate of both diseases recovered. Moreover, we considered the individuals in the asymptomatic COVID-19 compartment ($A^C$) leave to infected COVID-19 compartment ($I^C$) at a rate of asymptomatic COVID-19 infected people $\alpha _2$, and to both diseases infection a force of infection $\epsilon \lambda ^T$ and some component is the recovery rate of asymptomatic COVID-19 infected people $\omega _2$.

Similarly, the individuals of the COVID-19 disease infection ($I^C$) become recovered from COVID-19 at a rate of $\rho _2$ or shifted to both diseases infection ($I^{TC}$) and both diseases are infectious at a rate of $\theta _2$ and $d_C$ respectively, COVID-19 disease death rate in this compartment. In addition, the recovered from COVID-19 ($R^C$) has the chance to leave either compartment ($I^{RT}$), respectively, at a rate of $\nu _2$. Then both latent COVID-19 and TB co-infected individuals ($I^{RT}$) move to the compartment (*R*) at a recovery rate from COVID-19 and TB sequentially. The latent co-infection diseases population in the compartment ($L^{TC}$) either progresses to the co-infection ($L^{TC}$) at a rate $\alpha _{12}$. The remaining component is assumed to be shifted to either compartment at a $\sigma$ as illustrated in Fig. 1. That is, the susceptible people in the compartment ($L^{TC}$) move to ($I^T$) with a rate of recovery in COVID-19 people $p_2^{\sigma }$, move to the $I^C$ with a rate of recovery in TB infected people of $p_1^{\sigma }$, and become recovered at a rate of $(1 - (p_1 + p_2))\sigma$. Moreover, we considered that both diseases dual infection $I^{TC}$ leave compartments ($I^T, I^C,$ and *R*) denoted at a rate of $m_1 \tau , m_2 \tau ,$ or $(1 - (m_1+m_2))\tau$ while the co-infection induced death rate is $d_{TC}$. Finally, recovered from both TB and COVID-19 (*R*) at the rate of natural death is denoted by $\mu$.

## Existence and uniqueness results

In this segment, we utilize the fixed-point procedure to present the existence and uniqueness of the solution for the proposed model. Applying the Atangana-Baleanu fractal-fractional integral operator on the model (1) and utilizing the initial conditions, we obtain

2 $$\begin{aligned} S^{TC}(s)&=S^{TC}(0)+\frac{k_1k_2}{\mathscr{A}\mathscr{B}(k_1)\Gamma (k_1)}\int _{0}^{s}u^{k_2-1}(s-u)^{k_1-1}\Big [\pi -(\lambda ^{T}+\lambda ^{C}+\mu )S^{TC}\Big ]du\nonumber \\&\quad +\frac{k_2(1-k_1)s^{k_2-1}}{\mathscr{A}\mathscr{B}(k_1)}\Big [\pi -(\lambda ^{T}+\lambda ^{C}+\mu )S^{TC}\Big ], \end{aligned}$$

3 $$\begin{aligned} L^{T}(s)&=L^{T}(0)+\frac{k_1k_2}{\mathscr{A}\mathscr{B}(k_1)\Gamma (k_1)}\int _{0}^{s}u^{k_2-1}(s-u)^{k_1-1}\Big [\lambda ^{T} S^{TC}-(\alpha _{1}+\omega _{1}+\eta \lambda ^{C}+\mu ) L^{T}\Big ]du\nonumber \\&\quad +\frac{k_2(1-k_1)s^{k_2-1}}{\mathscr{A}\mathscr{B}(k_1)}\Big [\lambda ^{T} S^{TC}-(\alpha _{1}+\omega _{1}+\eta \lambda ^{C}+\mu ) L^{T}\Big ], \end{aligned}$$

4 $$\begin{aligned} I^{T}(s)&=I^{T}(0)+\frac{k_1k_2}{\mathscr{A}\mathscr{B}(k_1)\Gamma (k_1)}\int _{0}^{s}u^{k_2-1}(s-u)^{k_1-1}\Big [\alpha _1 L^{T}+ p_1^{\sigma } L^{TC}+m_1 \tau I^{TC}-(\rho _1+\theta _1+\mu +d^{T})I^{T}\Big ]du\nonumber \\&\quad +\frac{k_2(1-k_1)s^{k_2-1}}{\mathscr{A}\mathscr{B}(k_1)}\Big [\alpha _1 L^{T}+ p_1^{\sigma } L^{TC}+m_1 \tau I^{TC}-(\rho _1+\theta _1+\mu +d^{T})I^{T}\Big ], \end{aligned}$$

5 $$\begin{aligned} R^{T}(s)&=R^{T}(0)+\frac{k_1k_2}{\mathscr{A}\mathscr{B}(k_1)\Gamma (k_1)}\int _{0}^{s}u^{k_2-1}(s-u)^{k_1-1}\Big [\omega _1 L^{T}+\rho _1 I^{T}-(\nu _1+\mu ) R^{T}\Big ]du\nonumber \\&\quad +\frac{k_2(1-k_1)s^{k_2-1}}{\mathscr{A}\mathscr{B}(k_1)}\Big [\omega _1 L^{T}+\rho _1 I^{T}-(\nu _1+\mu ) R^{T}\Big ], \end{aligned}$$

6 $$\begin{aligned} I^{RC}(s)&=I^{RC}(0)+\frac{k_1k_2}{\mathscr{A}\mathscr{B}(k_1)\Gamma (k_1)}\int _{0}^{s}u^{k_2-1}(s-u)^{k_1-1}\Big [\nu _1 R^{T}-(\beta _1+\mu +d^C) I^{RC}\Big ]du\nonumber \\&\quad +\frac{k_2(1-k_1)s^{k_2-1}}{\mathscr{A}\mathscr{B}(k_1)}\Big [\nu _1 R^{T}-(\beta _1+\mu +d^C) I^{RC}\Big ], \end{aligned}$$

7 $$\begin{aligned} A^{C}(s)&=A^{C}(0)+\frac{k_1k_2}{\mathscr{A}\mathscr{B}(k_1)\Gamma (k_1)}\int _{0}^{s}u^{k_2-1}(s-u)^{k_1-1}\Big [\lambda ^T S^{TC}-(\alpha _2+\omega _2+\epsilon \lambda ^T +\mu ) A^C\Big ]du\nonumber \\&\quad +\frac{k_2(1-k_1)s^{k_2-1}}{\mathscr{A}\mathscr{B}(k_1)}\Big [\lambda ^C S^{TC}-(\alpha _2+\omega _2+\epsilon \lambda ^T +\mu ) A^C\Big ], \end{aligned}$$

8 $$\begin{aligned} I^{C}(s)&=I^{C}(0)+\frac{k_1k_2}{\mathscr{A}\mathscr{B}(k_1)\Gamma (k_1)}\int _{0}^{s}u^{k_2-1}(s-u)^{k_1-1}\Big [\alpha _2 A^C+ p_2^{\sigma } L^{TC}+m_2 \tau I^{TC}+ r R^C-(\rho _2+\theta _2+\mu +d^C)I^C\Big ]du\nonumber \\&\quad +\frac{k_2(1-k_1)s^{k_2-1}}{\mathscr{A}\mathscr{B}(k_1)}\Big [\alpha _2 A^C+ p_2^{\sigma } L^{TC}+m_2 \tau I^{TC}+ r R^C-(\rho _2+\theta _2+\mu +d^C)I^C\Big ], \end{aligned}$$

9 $$\begin{aligned} R^{C}(s)&=R^{C}(0)+\frac{k_1k_2}{\mathscr{A}\mathscr{B}(k_1)\Gamma (k_1)}\int _{0}^{s}u^{k_2-1}(s-u)^{k_1-1}\Big [\omega _2 A^C+\rho _2 I^C-(\nu _2 +r+\mu )R^C\Big ]du\nonumber \\&\quad +\frac{k_2(1-k_1)s^{k_2-1}}{\mathscr{A}\mathscr{B}(k_1)}\Big [\omega _2 A^C+\rho _2 I^C-(\nu _2 +r+\mu )R^C\Big ], \end{aligned}$$

10 $$\begin{aligned} I^{RT}(s)&=I^{RT}(0)+\frac{k_1k_2}{\mathscr{A}\mathscr{B}(k_1)\Gamma (k_1)}\int _{0}^{s}u^{k_2-1}(s-u)^{k_1-1}\Big [\nu _2 R^C-(\beta _2+\mu +d^T) I^{RT}\Big ]du\nonumber \\&\quad +\frac{k_2(1-k_1)s^{k_2-1}}{\mathscr{A}\mathscr{B}(k_1)}\Big [\nu _2 R^C-(\beta _2+\mu +d^T) I^{RT}\Big ], \end{aligned}$$

11 $$\begin{aligned} L^{TC}(s)&=L^{TC}(0)+\frac{k_1k_2}{\mathscr{A}\mathscr{B}(k_1)\Gamma (k_1)}\int _{0}^{s}u^{k_2-1}(s-u)^{k_1-1}\Big [\eta \lambda ^C L^T+\epsilon \lambda ^T A^C- (\alpha _{12}+\sigma +\mu ) L^{TC}\Big ]du \qquad \qquad \nonumber \\&\quad +\frac{k_2(1-k_1)s^{k_2-1}}{\mathscr{A}\mathscr{B}(k_1)}\Big [\eta \lambda ^C L^T+\epsilon \lambda ^T A^C- (\alpha _{12}+\sigma +\mu ) L^{TC}\Big ], \end{aligned}$$

12 $$\begin{aligned} I^{TC}(s)&=I^{TC}(0)+\frac{k_1k_2}{\mathscr{A}\mathscr{B}(k_1)\Gamma (k_1)}\int _{0}^{s}u^{k_2-1}(s-u)^{k_1-1}\Big [\alpha _{12} L^{TC}+ \theta _1 I^T+ \theta _2 I^C - (\tau +\mu +d^{TC}) I^{TC}\Big ]du\qquad \qquad \nonumber \\&\quad +\frac{k_2(1-k_1)s^{k_2-1}}{\mathscr{A}\mathscr{B}(k_1)}\Big [\alpha _{12} L^{TC}+ \theta _1 I^T+ \theta _2 I^C - (\tau +\mu +d^{TC}) I^{TC}\Big ], \end{aligned}$$

13 $$\begin{aligned} R(s)&=R(0)+\frac{k_1k_2}{\mathscr{A}\mathscr{B}(k_1)\Gamma (k_1)}\int _{0}^{s}u^{k_2-1}(s-u)^{k_1-1}\Big [\beta _1 I^{RC}+\beta _2 I^{RT}\nonumber \\&\quad +(1-(p_1+p_2 ))\sigma L^{TC}+(1-(m_1+m_2 ))\tau I^{TC}-\mu R\Big ]du+\frac{k_2(1-k_1)s^{k_2-1}}{\mathscr{A}\mathscr{B}(k_1)}\quad \qquad \qquad \qquad \qquad \nonumber \\&\quad \times \Big [\beta _1 I^{RC}+\beta _2 I^{RT}+(1-(p_1+p_2 ))\sigma L^{TC}+(1-(m_1+m_2 ))\tau I^{TC}-\mu R\Big ]. \end{aligned}$$

Let us consider the function $\mathscr {Q}_i$ for $i=1,2,...,12$ or $i \in \mathscr {N}^{12}_1$, thus

$$\begin{aligned} \begin{aligned} {\mathscr {Q}}_{1} \big (s,S^{TC} \big )&=\pi -\big (\lambda ^{T}+\lambda ^{C}+\mu \big )S^{TC},\\ {\mathscr {Q}}_{2}\big (s,L^{T}\big )&=\lambda ^{T} S^{TC}-\big (\alpha _{1}+\omega _{1}+\eta \lambda ^{C}+\mu \big ) L^{T},\\ {\mathscr {Q}}_{3}\big (s,I^{T}\big )&=\alpha _1 L^{T}+ p_1^{\sigma } L^{TC}+m_1 \tau I^{TC}-\big (\rho _1+\theta _1+\mu +d^{T}\big )I^{T},\\ {\mathscr {Q}}_{4}\big (s,R^{T}\big )&=\omega _1 L^{T}+\rho _1 I^{T}-\big (\nu _1+\mu \big ) R^{T},\\ {\mathscr {Q}}_{5}\big (s,I^{RC}\big )&=\nu _1 R^{T}-\big (\beta _1+\mu +d^C\big ) I^{RC},\\ {\mathscr {Q}}_{6}\big (s,A^C\big )&=\lambda ^C S^{TC}-\big (\alpha _2+\omega _2+\epsilon \lambda ^T +\mu \big ) A^C,\\ {\mathscr {Q}}_{7}\big (s,I^C\big )&=\alpha _2 A^C+ p_2^{\sigma } L^{TC}+m_2 \tau I^{TC}+ r R^C-\big (\rho _2+\theta _2+\mu +d^C\big )I^C,\\ {\mathscr {Q}}_{8}\big (s,R^C\big )&=\omega _2 A^C+\rho _2 I^C-\big (\nu _2 +r+\mu \big )R^C,\\ {\mathscr {Q}}_{9}\big (s,I^{RT}\big )&=\nu _2 R^C-\big (\beta _2+\mu +d^T\big ) I^{RT},\\ {\mathscr {Q}}_{10}\big (s,L^{TC}\big )&=\eta \lambda ^C L^T+\epsilon \lambda ^T A^C- \big (\alpha _{12}+ p_1^{\sigma }+p_2^{\sigma }+\big (1-\big (p_1+p_2 \big )\big )\sigma +\mu \big ) L^{TC},\\ {\mathscr {Q}}_{11}\big (s,I^{TC}\big )&=\alpha _{12} L^{TC}+ \theta _1 I^T+ \theta _2 I^C - \big (m_1 \tau +m_2\tau +\big (1-\big (m_1+m_2\big )\big )\tau +\mu +d^{TC}\big ) I^{TC},\\ {\mathscr {Q}}_{12}(s,R)&=\beta _1 I^{RC}+\beta _2 I^{RT}+\big (1-\big (p_1+p_2 \big )\big )\sigma L^{TC}+\big (1-\big (m_1+m_2 \big )\big )\tau I^{TC}-\mu R.\\ \end{aligned} \end{aligned}$$

($\mathscr {H}$:) For proving our results, we consider the following assumption: For the $S^{TC}(s)$, $\widetilde{S}^{TC}(s)$, $L^{T}(s)$, $\widetilde{L}^T(s)$, $I^T(s)$, $\widetilde{I}^T(s)$, $R^T(s)$, $\widetilde{R}^T(s)$, $I^{RC}(s)$, $\widetilde{I}^{RC}(s)$, $A^C(s)$, $\widetilde{A}^C(s)$, $I^C(s)$, $\widetilde{I}^C(s)$, $R^C(s)$, $\widetilde{R}^C(s)$, $I^{RT}(s)$, $\widetilde{I}^{RT}(s)$, $L^{TC}(s)$, $\widetilde{L}^{TC}(s)$, $I^{TC}(s)$, $\widetilde{I}^{TC}(s)$, *R*(*s*), $\widetilde{R}(s)\in \mathscr {L}[0, 1]$, be continuous function, such that $\Vert S^{TC}(s)\Vert \le \mathscr {L}_1$, $\Vert L^{T}(s)\Vert \le \mathscr {L}_2$, $\Vert I^T(s)\Vert \le \mathscr {L}_3$, $\Vert R^T(s)\Vert \le \mathscr {L}_4$, $\Vert I^{RC}(s)\Vert \le \mathscr {L}_5$, $\Vert A^C(s)\Vert \le \mathscr {L}_6$, $\Vert I^C(s)\Vert \le \mathscr {L}_7$, $\Vert R^C(s)\Vert \le \mathscr {L}_8$, $\Vert I^{RT}(s)\Vert \le \mathscr {L}_9$, $\Vert L^{TC}(s)\Vert \le \mathscr {L}_{10}$, $\Vert I^{TC}(s)\Vert \le \mathscr {L}_{11}$, $\Vert R(s)\Vert \le \mathscr {L}_{12}$ for non-negative constant $\mathscr {L}_1$, $\mathscr {L}_2$, $\mathscr {L}_3$, $\mathscr {L}_4$, $\mathscr {L}_5$, $\mathscr {L}_6$, $\mathscr {L}_7$, $\mathscr {L}_8$, $\mathscr {L}_9$, $\mathscr {L}_{10}$, $\mathscr {L}_{11}$, $\mathscr {L}_{12}>0$.

### Theorem 1

The Lipschitz condition is satisfy the $\mathscr {Q}_i$ for $i \in \mathscr {N}^{12}_1$, if the assumption $\mathscr {H}$ is holds true and fulfills and $\Psi _i<1$, for $i \in \mathscr {N}^{12}_1$.

### Proof

Now, we prove that ${\mathscr {Q}}_{1}(s,S^{TC})$ fulfills the Lipschitz condition. For $S^{TC}(s)$, $\widetilde{S}^{TC}(s)$, we get

$$\begin{aligned} \left\| \mathscr {Q}_1(s,S^{TC})-\mathscr {Q}_1(s,\widetilde{S}^{TC})\right\|&=\Big |\Big |\pi -(\lambda ^{T}+\lambda ^{C}+\mu )S^{TC}-\left( \pi -(\lambda ^{T}+\lambda ^{C}+\mu )\widetilde{S}^{TC}\right) \Big |\Big |,\\&\le \left( \lambda ^{T}+\lambda ^{C}+\mu \right) \Vert S^{TC} - \widetilde{S}^{TC}\Vert ,\\&\le \Psi _1 \Vert S^{TC} - \widetilde{S}^{TC}\Vert , \end{aligned}$$

where, $\Psi _1=\lambda ^{T}+\lambda ^{C}+\mu$. Hence, $\mathscr {Q}_1$ satisfies the Lipschitz condition with Lipschitz constant $\Psi _1$. Similarly, the other kernels satisfy the Lipschitz condition as follows:

$$\begin{aligned} \begin{aligned} \left\| \mathscr {Q}_2(s,L^{T})-\mathscr {Q}_2(s,\widetilde{L}^{T})\right\|&\le \Psi _2 \Vert L^{T} - \widetilde{L}^{T}\Vert , \\ \left\| \mathscr {Q}_3(s,I^{T})-\mathscr {Q}_3(s,\widetilde{I}^{T})\right\|&\le \Psi _3 \Vert I^{T} - \widetilde{I}^{T}\Vert ,\\ \left\| \mathscr {Q}_4(s,R^{T})-\mathscr {Q}_4(s,\widetilde{R}^{T})\right\|&\le \Psi _4 \Vert R^{T} - \widetilde{R}^{T}\Vert , \\ \left\| \mathscr {Q}_5(s,I^{RC})-\mathscr {Q}_5(s,\widetilde{I}^{RC})\right\|&\le \Psi _5 \Vert I^{RC} - \widetilde{I}^{RC}\Vert ,\\ \left\| \mathscr {Q}_6(s,A^{C})-\mathscr {Q}_6(s,\widetilde{A}^{C})\right\|&\le \Psi _6 \Vert A^{C} - \widetilde{A}^{C}\Vert , \\ \left\| \mathscr {Q}_7(s,I^{C})-\mathscr {Q}_7(s,\widetilde{I}^{C})\right\|&\le \Psi _7 \Vert I^{C} - \widetilde{I}^{C}\Vert ,\\ \left\| \mathscr {Q}_8(s,R^{C})-\mathscr {Q}_8(s,\widetilde{R}^{C})\right\|&\le \Psi _8 \Vert R^{C} - \widetilde{R}^{C}\Vert , \\ \left\| \mathscr {Q}_9(s,I^{RT})-\mathscr {Q}_9(s,\widetilde{I}^{RT})\right\|&\le \Psi _9 \Vert I^{RT} - \widetilde{I}^{RT}\Vert ,\\ \left\| \mathscr {Q}_{10}(s,L^{TC})-\mathscr {Q}_{10}(s,\widetilde{L}^{TC})\right\|&\le \Psi _{10} \Vert L^{TC} - \widetilde{L}^{TC}\Vert , \\ \left\| \mathscr {Q}_{11}(s,I^{TC})-\mathscr {Q}_{11}(s,\widetilde{I}^{TC})\right\|&\le \Psi _{11} \Vert I^{TC} - \widetilde{I}^{TC}\Vert ,\\ \left\| \mathscr {Q}_{12}(s,R)-\mathscr {Q}_{12}(s,\widetilde{R})\right\|&\le \Psi _{12} \Vert R - \widetilde{R}\Vert .\\ \end{aligned} \end{aligned}$$

Hence, all the kernels $\mathscr {Q}_i$, $i \in \mathscr {N}^{12}_1$ satisfies the Lipschitz property with constant $\Psi _i<1$ for $i \in \mathscr {N}^{12}_1$. The proof is completed. $\square$

Now, Eqs. (2) to (13) can be rewrite as follows:

14 $$\begin{aligned} S^{TC}(s)&=S^{TC}(0)+\frac{k_1k_2}{\mathscr{A}\mathscr{B}(k_1)\Gamma (k_1)}\int _{0}^{s}u^{k_2-1}(s-u)^{k_1-1}\mathscr {Q}_1(u,S^{TC}(u))du+\frac{k_2(1-k_1)}{\mathscr{A}\mathscr{B}(k_1)}s^{k_2-1}\mathscr {Q}_1(s,S^{TC}(s)), \end{aligned}$$

15 $$\begin{aligned} L^{T}(s)&=L^{T}(0)+\frac{k_1k_2}{\mathscr{A}\mathscr{B}(k_1)\Gamma (k_1)}\int _{0}^{s}u^{k_2-1}(s-u)^{k_1-1}\mathscr {Q}_2(u,L^{T}(u))du+\frac{k_2(1-k_1)}{\mathscr{A}\mathscr{B}(k_1)}s^{k_2-1}\mathscr {Q}_2(s,L^{T}(s)), \end{aligned}$$

16 $$\begin{aligned} I^{T}(s)&=I^{T}(0)+\frac{k_1k_2}{\mathscr{A}\mathscr{B}(k_1)\Gamma (k_1)}\int _{0}^{s}u^{k_2-1}(s-u)^{k_1-1}\mathscr {Q}_3(u,I^{T}(u))du+\frac{k_2(1-k_1)}{\mathscr{A}\mathscr{B}(k_1)}s^{k_2-1}\mathscr {Q}_3(s,I^{T}(s)), \end{aligned}$$

17 $$\begin{aligned} R^{T}(s)&=R^{T}(0)+\frac{k_1k_2}{\mathscr{A}\mathscr{B}(k_1)\Gamma (k_1)}\int _{0}^{s}u^{k_2-1}(s-u)^{k_1-1}\mathscr {Q}_4(u,R^{T}(u))du+\frac{k_2(1-k_1)}{\mathscr{A}\mathscr{B}(k_1)}s^{k_2-1}\mathscr {Q}_4(s,R^{T}(s)), \end{aligned}$$

18 $$\begin{aligned} I^{RC}(s)&=I^{RC}(0)+\frac{k_1k_2}{\mathscr{A}\mathscr{B}(k_1)\Gamma (k_1)}\int _{0}^{s}u^{k_2-1}(s-u)^{k_1-1}\mathscr {Q}_5(u,I^{RC}(u))du+\frac{k_2(1-k_1)}{\mathscr{A}\mathscr{B}(k_1)}s^{k_2-1}\mathscr {Q}_5(s,I^{RC}(s)), \end{aligned}$$

19 $$\begin{aligned} A^{C}(s)&=A^{C}(0)+\frac{k_1k_2}{\mathscr{A}\mathscr{B}(k_1)\Gamma (k_1)}\int _{0}^{s}u^{k_2-1}(s-u)^{k_1-1}\mathscr {Q}_6(u,A^{C}(u))du+\frac{k_2(1-k_1)}{\mathscr{A}\mathscr{B}(k_1)}s^{k_2-1}\mathscr {Q}_6(s,A^{C}(s)), \end{aligned}$$

20 $$\begin{aligned} I^{C}(s)&=I^{C}(0)+\frac{k_1k_2}{\mathscr{A}\mathscr{B}(k_1)\Gamma (k_1)}\int _{0}^{s}u^{k_2-1}(s-u)^{k_1-1}\mathscr {Q}_7(u,I^{C}(u))du+\frac{k_2(1-k_1)}{\mathscr{A}\mathscr{B}(k_1)}s^{k_2-1}\mathscr {Q}_7(s,I^{C}(s)), \end{aligned}$$

21 $$\begin{aligned} R^{C}(s)&=R^{C}(0)+\frac{k_1k_2}{\mathscr{A}\mathscr{B}(k_1)\Gamma (k_1)}\int _{0}^{s}u^{k_2-1}(s-u)^{k_1-1}\mathscr {Q}_8(u,R^{C}(u))du+\frac{k_2(1-k_1)}{\mathscr{A}\mathscr{B}(k_1)}s^{k_2-1}\mathscr {Q}_8(s,R^{C}(s)), \end{aligned}$$

22 $$\begin{aligned} I^{RT}(s)&=I^{RT}(0)+\frac{k_1k_2}{\mathscr{A}\mathscr{B}(k_1)\Gamma (k_1)}\int _{0}^{s}u^{k_2-1}(s-u)^{k_1-1}\mathscr {Q}_9(u,I^{RT}(u))du+\frac{k_2(1-k_1)}{\mathscr{A}\mathscr{B}(k_1)}s^{k_2-1}\mathscr {Q}_9(s,I^{RT}(s)), \end{aligned}$$

23 $$\begin{aligned} L^{TC}(s)&=L^{TC}(0)+\frac{k_1k_2}{\mathscr{A}\mathscr{B}(k_1)\Gamma (k_1)}\int _{0}^{s}u^{k_2-1}(s-u)^{k_1-1}\mathscr {Q}_{10}(u,L^{TC}(u))du+\frac{k_2(1-k_1)}{\mathscr{A}\mathscr{B}(k_1)}s^{k_2-1}\mathscr {Q}_{10}(s,L^{TC}(s)), \end{aligned}$$

24 $$\begin{aligned} I^{TC}(s)&=I^{TC}(0)+\frac{k_1k_2}{\mathscr{A}\mathscr{B}(k_1)\Gamma (k_1)}\int _{0}^{s}u^{k_2-1}(s-u)^{k_1-1}\mathscr {Q}_{11}(u,I^{TC}(u))du+\frac{k_2(1-k_1)}{\mathscr{A}\mathscr{B}(k_1)}s^{k_2-1}\mathscr {Q}_{11}(s,I^{TC}(s)), \end{aligned}$$

25 $$\begin{aligned} R(s)&=R(0)+\frac{k_1k_2}{\mathscr{A}\mathscr{B}(k_1)\Gamma (k_1)}\int _{0}^{s}u^{k_2-1}(s-u)^{k_1-1}\mathscr {Q}_{12}(u,R(u))du+\frac{k_2(1-k_1)}{\mathscr{A}\mathscr{B}(k_1)}s^{k_2-1}\mathscr {Q}_{12}(s,R(s)), \end{aligned}$$

together with initial conditions are given as

$$\begin{aligned} \begin{aligned} S^{TC}_0(s)&=S^{TC}(0), L^{T}_0(s)=L^{T}(0), I^{T}_0(s)=I^{T}(0), R^{T}_0(s)=R^{T}(0), I^{RC}_0(s)=I^{RC}(0), A^{C}_0(s)=A^{C}(0), I^{C}_0(s)=I^{C}(0),\\ R^{C}_0(s)&=R^{C}(0), I^{RT}_0(s)=I^{RT}(0), L^{TC}_0(s)=L^{TC}(0), I^{TC}_0(s)=I^{TC}(0), R_0(s)=R(0). \end{aligned} \end{aligned}$$

Now, we define the recursive formulas for the Eqs. (14)–(25) as follows:

$$\begin{aligned} S_n^{TC}(s)&=S^{TC}(0)+\frac{k_1k_2}{\mathscr{A}\mathscr{B}(k_1)\Gamma (k_1)}\int _{0}^{s}u^{k_2-1}(s-u)^{k_1-1}\mathscr {Q}_1(u,S_{n-1}^{TC}(u))du+\frac{k_2(1-k_1)}{\mathscr{A}\mathscr{B}(k_1)}s^{k_2-1}\mathscr {Q}_1(s,S_{n-1}^{TC}(s)),\\ L_n^{T}(s)&=L^{T}(0)+\frac{k_1k_2}{\mathscr{A}\mathscr{B}(k_1)\Gamma (k_1)}\int _{0}^{s}u^{k_2-1}(s-u)^{k_1-1}\mathscr {Q}_2(u,L_{n-1}^{T}(u))du+\frac{k_2(1-k_1)}{\mathscr{A}\mathscr{B}(k_1)}s^{k_2-1}\mathscr {Q}_2(s,L_{n-1}^{T}(s)), \\ I_n^{T}(s)&=I^{T}(0)+\frac{k_1k_2}{\mathscr{A}\mathscr{B}(k_1)\Gamma (k_1)}\int _{0}^{s}u^{k_2-1}(s-u)^{k_1-1}\mathscr {Q}_3(u,I_{n-1}^{T}(u))du+\frac{k_2(1-k_1)}{\mathscr{A}\mathscr{B}(k_1)}s^{k_2-1}\mathscr {Q}_3(s,I_{n-1}^{T}(s)), \\ R_n^{T}(s)&=R^{T}(0)+\frac{k_1k_2}{\mathscr{A}\mathscr{B}(k_1)\Gamma (k_1)}\int _{0}^{s}u^{k_2-1}(s-u)^{k_1-1}\mathscr {Q}_4(u,R_{n-1}^{T}(u))du+\frac{k_2(1-k_1)}{\mathscr{A}\mathscr{B}(k_1)}s^{k_2-1}\mathscr {Q}_4(s,R_{n-1}^{T}(s)), \\ I_n^{RC}(s)&=I^{RC}(0)+\frac{k_1k_2}{\mathscr{A}\mathscr{B}(k_1)\Gamma (k_1)}\int _{0}^{s}u^{k_2-1}(s-u)^{k_1-1}\mathscr {Q}_5(u,I_{n-1}^{RC}us))du+\frac{k_2(1-k_1)}{\mathscr{A}\mathscr{B}(k_1)}s^{k_2-1}\mathscr {Q}_5(s,I_{n-1}^{RC}(s)), \\ A_n^{C}(s)&=A^{C}(0)+\frac{k_1k_2}{\mathscr{A}\mathscr{B}(k_1)\Gamma (k_1)}\int _{0}^{s}u^{k_2-1}(s-u)^{k_1-1}\mathscr {Q}_6(u,A_{n-1}^{C}(u))du+\frac{k_2(1-k_1)}{\mathscr{A}\mathscr{B}(k_1)}s^{k_2-1}\mathscr {Q}_6(s,A_{n-1}^{C}(s)), \\ I_n^{C}(s)&=I^{C}(0)+\frac{k_1k_2}{\mathscr{A}\mathscr{B}(k_1)\Gamma (k_1)}\int _{0}^{s}u^{k_2-1}(s-u)^{k_1-1}\mathscr {Q}_7(u,I_{n-1}^{C}(u))du+\frac{k_2(1-k_1)}{\mathscr{A}\mathscr{B}(k_1)}s^{k_2-1}\mathscr {Q}_7(s,I_{n-1}^{C}(s)), \\ R_n^{C}(s)&=R^{C}(0)+\frac{k_1k_2}{\mathscr{A}\mathscr{B}(k_1)\Gamma (k_1)}\int _{0}^{s}u^{k_2-1}(s-u)^{k_1-1}\mathscr {Q}_8(u,R_{n-1}^{C}(u))du+\frac{k_2(1-k_1)}{\mathscr{A}\mathscr{B}(k_1)}s^{k_2-1}\mathscr {Q}_8(s,R_{n-1}^{C}(s)), \\ I_n^{RT}(s)&=I^{RT}(0)+\frac{k_1k_2}{\mathscr{A}\mathscr{B}(k_1)\Gamma (k_1)}\int _{0}^{s}u^{k_2-1}(s-u)^{k_1-1}\mathscr {Q}_9(u,I_{n-1}^{RT}(u))du+\frac{k_2(1-k_1)}{\mathscr{A}\mathscr{B}(k_1)}s^{k_2-1}\mathscr {Q}_9(s,I_{n-1}^{RT}(s)), \\ L_n^{TC}(s)&=L^{TC}(0)+\frac{k_1k_2}{\mathscr{A}\mathscr{B}(k_1)\Gamma (k_1)}\int _{0}^{s}u^{k_2-1}(s-u)^{k_1-1}\mathscr {Q}_{10}(u,L_{n-1}^{TC}(u))du+\frac{k_2(1-k_1)}{\mathscr{A}\mathscr{B}(k_1)}s^{k_2-1}\mathscr {Q}_{10}(s,L_{n-1}^{TC}(s)), \\ I_n^{TC}(s)&=I^{TC}(0)+\frac{k_1k_2}{\mathscr{A}\mathscr{B}(k_1)\Gamma (k_1)}\int _{0}^{s}u^{k_2-1}(s-u)^{k_1-1}\mathscr {Q}_{11}(u,I_{n-1}^{TC}(u))du+\frac{k_2(1-k_1)}{\mathscr{A}\mathscr{B}(k_1)}s^{k_2-1}\mathscr {Q}_{11}(s,I_{n-1}^{TC}(s)), \\ R_n(s)&=R(0)+\frac{k_1k_2}{\mathscr{A}\mathscr{B}(k_1)\Gamma (k_1)}\int _{0}^{s}u^{k_2-1}(s-u)^{k_1-1}\mathscr {Q}_{12}(u,R_{n-1}(u))du+\frac{k_2(1-k_1)}{\mathscr{A}\mathscr{B}(k_1)}s^{k_2-1}\mathscr {Q}_{12}(s,R_{n-1}(s)). \end{aligned}$$

### Theorem 2

The model (1) has a solution if the following are holds true:

$$\begin{aligned} \Delta = \max {\Psi _i} < 1,\ i \in \mathbb {N}_1^{12}. \end{aligned}$$

### Proof

We define the functions as follows:

$$\begin{aligned} \begin{aligned} \mathscr {U}1_{n}(s)&=S_{n+1}^{TC}(s)-S^{TC}(s),\ \mathscr {U}2_{n}(s)=L_{n+1}^{T}(s)-L^{T}(s), \ \mathscr {U}3_{n}(s)=I_{n+1}^{T}(s)-I^{T}(s),\\ \mathscr {U}4_{n}(s)&=R_{n+1}^{T}(s)-R^{T}(s), \ \mathscr {U}5_{n}(s)=I_{n+1}^{RC}(s)-I^{RC}(s),\ \mathscr {U}6_{n}(s)=A_{n+1}^{C}(s)-A^{C}(s), \\ \mathscr {U}7_{n}(s)&=I_{n+1}^{C}(s)-I^{C}(s),\ \mathscr {U}8_{n}(s)=R_{n+1}^{C}(s)-R^{C}(s), \ \mathscr {U}9_{n}(s)=I_{n+1}^{RT}(s)-I^{RT}(s),\\ \mathscr {U}10_{n}(s)&=L_{n+1}^{TC}(s)-L^{TC}(s),\ \mathscr {U}11_{n}(s)=I_{n+1}^{TC}(s)-I^{TC}(s), \ \mathscr {U}12_{n}(s)=R_{n+1}(s)-R(s). \end{aligned} \end{aligned}$$

Then, we find that

$$\begin{aligned} \begin{aligned} \Big \Vert \mathscr {U}1_n(s)\Big \Vert&= \frac{k_1k_2}{\mathscr{A}\mathscr{B}(k_1)\Gamma (k_1)}\int _{0}^{s}u^{k_2-1}(s-u)^{k_1-1}\Big \Vert \mathscr {Q}_{1}(u,S_{n}^{TC}(u))-\mathscr {Q}_{1}(u,S_{n}^{TC}(u))\Big \Vert du\\&\qquad +\frac{k_2(1-k_1)s^{k_2-1}}{\mathscr{A}\mathscr{B}(k_1)}\Big \Vert \mathscr {Q}_{1}(s,S_{n_1}^{TC}(s))-\mathscr {Q}_{1}(s,S^{TC}(s))\Big \Vert ,\\&\le \left[ \frac{k_1k_2\Gamma (k_2)}{\mathscr{A}\mathscr{B}(k_1)\Gamma (k_1+k_2)}+\frac{k_2(1-k_1)}{\mathscr{A}\mathscr{B}(k_1)}\right] \Psi _1 \Vert S_n^{TC}-S^{TC}\Vert ,\\&\le \left[ \frac{k_1k_2\Gamma (k_2)}{\mathscr{A}\mathscr{B}(k_1)\Gamma (k_1+k_2)}+\frac{k_2(1-k_1)}{\mathscr{A}\mathscr{B}(k_1)}\right] ^n \Psi _1^n \Vert S_1^{TC}-S^{TC}\Vert . \end{aligned} \end{aligned}$$

Similarly, we have

$$\begin{aligned} \Big \Vert \mathscr {U}2_n(s)\Big \Vert&\le \left[ \frac{k_1k_2\Gamma (k_2)}{\mathscr{A}\mathscr{B}(k_1)\Gamma (k_1+k_2)}+\frac{k_2(1-k_1)}{\mathscr{A}\mathscr{B}(k_1)}\right] ^n\Psi _2^n \Vert L_1^{T}-L^{T}\Vert , \\ \Big \Vert \mathscr {U}3_n(s)\Big \Vert&\le \left[ \frac{k_1k_2\Gamma (k_2)}{\mathscr{A}\mathscr{B}(k_1)\Gamma (k_1+k_2)}+\frac{k_2(1-k_1)}{\mathscr{A}\mathscr{B}(k_1)}\right] ^n\Psi _3^n \Vert I_1^{T}-I^{T}\Vert , \\ \Big \Vert \mathscr {U}4_n(s)\Big \Vert&\le \left[ \frac{k_1k_2\Gamma (k_2)}{\mathscr{A}\mathscr{B}(k_1)\Gamma (k_1+k_2)}+\frac{k_2(1-k_1)}{\mathscr{A}\mathscr{B}(k_1)}\right] ^n\Psi _4^n \Vert R_1^{T}-R^{T}\Vert , \\ \Big \Vert \mathscr {U}5_n(s)\Big \Vert&\le \left[ \frac{k_1k_2\Gamma (k_2)}{\mathscr{A}\mathscr{B}(k_1)\Gamma (k_1+k_2)}+\frac{k_2(1-k_1)}{\mathscr{A}\mathscr{B}(k_1)}\right] ^n\Psi _5^n \Vert I_1^{RC}-I^{RC}\Vert , \\ \Big \Vert \mathscr {U}6_n(s)\Big \Vert&\le \left[ \frac{k_1k_2\Gamma (k_2)}{\mathscr{A}\mathscr{B}(k_1)\Gamma (k_1+k_2)}+\frac{k_2(1-k_1)}{\mathscr{A}\mathscr{B}(k_1)}\right] ^n\Psi _6^n \Vert A_1^{C}-A^{C}\Vert , \\ \Big \Vert \mathscr {U}7_n(s)\Big \Vert&\le \left[ \frac{k_1k_2\Gamma (k_2)}{\mathscr{A}\mathscr{B}(k_1)\Gamma (k_1+k_2)}+\frac{k_2(1-k_1)}{\mathscr{A}\mathscr{B}(k_1)}\right] ^n\Psi _7^n \Vert I_1^{C}-I^{C}\Vert , \\ \Big \Vert \mathscr {U}8_n(s)\Big \Vert&\le \left[ \frac{k_1k_2\Gamma (k_2)}{\mathscr{A}\mathscr{B}(k_1)\Gamma (k_1+k_2)}+\frac{k_2(1-k_1)}{\mathscr{A}\mathscr{B}(k_1)}\right] ^n\Psi _8^n \Vert R_1^{C}-R^{C}\Vert , \\ \Big \Vert \mathscr {U}9_n(s)\Big \Vert&\le \left[ \frac{k_1k_2\Gamma (k_2)}{\mathscr{A}\mathscr{B}(k_1)\Gamma (k_1+k_2)}+\frac{k_2(1-k_1)}{\mathscr{A}\mathscr{B}(k_1)}\right] ^n\Psi _9^n \Vert I_1^{RT}-I^{RT}\Vert , \\ \Big \Vert \mathscr {U}10_n(s)\Big \Vert&\le \left[ \frac{k_1k_2\Gamma (k_2)}{\mathscr{A}\mathscr{B}(k_1)\Gamma (k_1+k_2)}+\frac{k_2(1-k_1)}{\mathscr{A}\mathscr{B}(k_1)}\right] ^n\Psi _{10}^n \Vert L_1^{TC}-L^{TC}\Vert , \\ \Big \Vert \mathscr {U}11_n(s)\Big \Vert&\le \left[ \frac{k_1k_2\Gamma (k_2)}{\mathscr{A}\mathscr{B}(k_1)\Gamma (k_1+k_2)}+\frac{k_2(1-k_1)}{\mathscr{A}\mathscr{B}(k_1)}\right] ^n\Psi _{11}^n \Vert I_1^{TC}-I^{TC}\Vert , \\ \Big \Vert \mathscr {U}12_n(s)\Big \Vert&\le \left[ \frac{k_1k_2\Gamma (k_2)}{\mathscr{A}\mathscr{B}(k_1)\Gamma (k_1+k_2)}+\frac{k_2(1-k_1)}{\mathscr{A}\mathscr{B}(k_1)}\right] ^n\Psi _{12}^n \Vert R_1-R\Vert . \end{aligned}$$

Thus, from the above twelve functions, when $n\rightarrow \infty$, then $\mathscr {U}(s)_{i_{n}}\rightarrow 0,$ for $i \in \mathscr {N}^{12}_1$ for $\Psi _i<1$, $(i=1,2,...,12)$ which completes the proof. $\square$

### Theorem 3

Due to assumption $\mathscr {H}$, the model (1) has unique solution if

$$\begin{aligned} \left[ \frac{k_1k_2\Gamma (k_2)}{\mathscr{A}\mathscr{B}(k_1)\Gamma (k_1+k_2)}+\frac{k_2(1-k_1)}{\mathscr{A}\mathscr{B}(k_1)}\right] \Psi _i\le 1,\ for\ i = 1, 2,...,12. \end{aligned}$$

### Proof

We assume that another existing solution $\Big (\widetilde{S}^{TC}(s),\ \widetilde{L}^{T}(s),\ \widetilde{I}^{T}(s),\ \widetilde{R}^{T}(s),\ \widetilde{I}^{RC}(s),\ \widetilde{A}^{C}(s),\ \widetilde{I}^{C}(s),\ \widetilde{R}^{C}(s),\ \widetilde{I}^{RT}(s),\ \widetilde{L}^{TC}(s), \widetilde{I}^{TC}(s),\ \widetilde{R}(s)\Big )$ with initial values, such that

$$\begin{aligned} \widetilde{S}^{TC}(s)=\widetilde{S}^{TC}(0)+\frac{k_1k_2}{\mathscr{A}\mathscr{B}(k_1)\Gamma (k_1)}\int _{0}^{s}u^{k_2-1}(s-u)^{k_1-1}\mathscr {Q}_{1}(u,\widetilde{S}^{TC}(u))du+\frac{k_2(1-k_1)s^{k_2-1}}{\mathscr{A}\mathscr{B}(k_1)}\mathscr {Q}_{1}(s,S^{TC}(s)). \end{aligned}$$

Now, we write

$$\begin{aligned} \Big \Vert S^{TC}-\widetilde{S}^{TC}\Big \Vert&= \frac{k_1k_2}{\mathscr{A}\mathscr{B}(k_1)\Gamma (k_1)}\int _{0}^{s}u^{k_2-1}(s-u)^{k_1-1}\Big \Vert \mathscr {Q}_{1}(u,S^{TC}(u))-\mathscr {Q}_{1}(u,\widetilde{S}^{TC}(u))\Big \Vert du\nonumber \\&\quad +\frac{k_2(1-k_1)s^{k_2-1}}{\mathscr{A}\mathscr{B}(k_1)}\Big \Vert \mathscr {Q}_{1}(s,S^{TC}(s))-\mathscr {Q}_{1}(s,\widetilde{S}^{TC}(s))\Big \Vert , \end{aligned}$$

and so

26 $$\begin{aligned} \left[ 1-\left[ \frac{k_1k_2\Gamma (k_2)}{\mathscr{A}\mathscr{B}(k_1)\Gamma (k_1+k_2)}+\frac{k_2(1-k_1)}{\mathscr{A}\mathscr{B}(k_1)}\right] \Psi _1\right] \Vert S^{TC}-\widetilde{S}^{TC}\Vert \le 0. \end{aligned}$$

The above inequality (26) is true if $\Vert S^{TC}-\widetilde{S}^{TC}\Vert = 0$, then consequently, $S^{TC}(s) = \widetilde{S}^{TC}(s)$. Hence the uniqueness of solution is proved. Similarly, we applying the same process yields $L^{T}$, $I^{T}$, $R^{T}$, $I^{RC}$, $A^{C}$, $I^{C}$, $R^{C}$, $I^{RT}$, $L^{TC}$, $I^{TC}$ and *R* can be proved. So, the model (1) has a unique solution. $\square$

## Ulam-Hyers stability of the proposed problem

In this segment, we obtain the Ulam-Hyers stability of the proposed model (1). We state the required definition.

### Definition 3

The model (1) has Ulam-Hyers stability if there exist constants $\mathscr {K}_i>0,\ i\in \mathbb {N}_1^{12}$ satisfying: For every $\varepsilon _i>0,\ i\in \mathbb {N}_1^{12}$, if

27 $$\begin{aligned} \left| {}^{{\mathscr {F}}{\mathscr {F}}{\mathscr {M}}}_{\text {0}}\mathscr {D}_{s}^{k_1,k_2}S^{TC}(s)-\mathscr {Q}_{1}(s,S^{TC})\right|&\le \varepsilon _1, \end{aligned}$$

28 $$\begin{aligned} \left| {}^{{\mathscr {F}}{\mathscr {F}}{\mathscr {M}}}_{\,\,\,\,\,\,\,\,\,\,\,\,\text {0}}\mathscr {D}_{s}^{k_1,k_2}L^{T}(s)-\mathscr {Q}_{2}(s,L^{T})\right|&\le \varepsilon _2, \end{aligned}$$

29 $$\begin{aligned} \left| {}^{{\mathscr {F}}{\mathscr {F}}{\mathscr {M}}}_{\,\,\,\,\,\,\,\,\,\,\,\,\text {0}}\mathscr {D}_{s}^{k_1,k_2}I^{T}(s)-\mathscr {Q}_{3}(s,I^{T})\right|&\le \varepsilon _3, \end{aligned}$$

30 $$\begin{aligned} \left| {}^{{\mathscr {F}}{\mathscr {F}}{\mathscr {M}}}_{\,\,\,\,\,\,\,\,\,\,\,\,\text {0}}\mathscr {D}_{s}^{k_1,k_2}R^{T}(s)-\mathscr {Q}_{4}(s,R^{T})\right|&\le \varepsilon _4, \end{aligned}$$

31 $$\begin{aligned} \left| {}^{{\mathscr {F}}{\mathscr {F}}{\mathscr {M}}}_{\,\,\,\,\,\,\,\,\,\,\,\,\text {0}}\mathscr {D}_{s}^{k_1,k_2}I^{RC}(s)-\mathscr {Q}_{5}(s,I^{RC})\right|&\le \varepsilon _5, \end{aligned}$$

32 $$\begin{aligned} \left| {}^{{\mathscr {F}}{\mathscr {F}}{\mathscr {M}}}_{\,\,\,\,\,\,\,\,\,\,\,\,\text {0}}\mathscr {D}_{s}^{k_1,k_2}A^{C}(s)-\mathscr {Q}_{6}(s,A^{C})\right|&\le \varepsilon _6, \end{aligned}$$

33 $$\begin{aligned} \left| {}^{{\mathscr {F}}{\mathscr {F}}{\mathscr {M}}}_{\,\,\,\,\,\,\,\,\,\,\,\,\text {0}}\mathscr {D}_{s}^{k_1,k_2}I^{C}(s)-\mathscr {Q}_{7}(s,I^{C})\right|&\le \varepsilon _7, \end{aligned}$$

34 $$\begin{aligned} \left| {}^{{\mathscr {F}}{\mathscr {F}}{\mathscr {M}}}_{\,\,\,\,\,\,\,\,\,\,\,\,\text {0}}\mathscr {D}_{s}^{k_1,k_2}R^{C}(s)-\mathscr {Q}_{8}(s,R^{C})\right|&\le \varepsilon _8, \end{aligned}$$

35 $$\begin{aligned} \left| {}^{{\mathscr {F}}{\mathscr {F}}{\mathscr {M}}}_{\,\,\,\,\,\,\,\,\,\,\,\,\text {0}}\mathscr {D}_{s}^{k_1,k_2}I^{RT}(s)-\mathscr {Q}_{9}(s,I^{RT})\right|&\le \varepsilon _9, \end{aligned}$$

36 $$\begin{aligned} \left| {}^{{\mathscr {F}}{\mathscr {F}}{\mathscr {M}}}_{\,\,\,\,\,\,\,\,\,\,\,\,\text {0}}\mathscr {D}_{s}^{k_1,k_2}L^{TC}(s)-\mathscr {Q}_{10}(s,L^{TC})\right|&\le \varepsilon _{10}, \end{aligned}$$

37 $$\begin{aligned} \left| {}^{{\mathscr {F}}{\mathscr {F}}{\mathscr {M}}}_{\,\,\,\,\,\,\,\,\,\,\,\,\text {0}}\mathscr {D}_{s}^{k_1,k_2}I^{TC}(s)-\mathscr {Q}_{11}(s,I^{TC})\right|&\le \varepsilon _{11}, \end{aligned}$$

38 $$\begin{aligned} \left| {}^{{\mathscr {F}}{\mathscr {F}}{\mathscr {M}}}_{\,\,\,\,\,\,\,\,\,\,\,\,\text {0}}\mathscr {D}_{s}^{k_1,k_2}R(s)-\mathscr {Q}_{12}(s,R)\right|&\le \varepsilon _{12}, \end{aligned}$$

and there exists a solution of the TB and COVID-19 model (1), $\widetilde{S}^{TC}(s)$, $\widetilde{L}^{T}(s)$, $\widetilde{I}^{T}(s)$, $\widetilde{R}^{T}(s)$, $\widetilde{I}^{RC}(s)$, $\widetilde{A}^{C}(s)$, $\widetilde{I}^{C}(s)$, $\widetilde{R}^{C}(s)$, $\widetilde{I}^{RT}(s)$, $\widetilde{L}^{TC}(s)$, $\widetilde{I}^{TC}(s)$ and $\widetilde{R}(s)$ that satisfying the given model, such that

$$\begin{aligned} \left\| S^{TC}-\widetilde{S}^{TC}\right\|&\le \mathscr {K}_1\varepsilon _1,\ \left\| L^{T}-\widetilde{L}^{T}\right\| \le \mathscr {K}_2\varepsilon _2,\ \left\| I^{T}-\widetilde{I}^{T}\right\| \le \mathscr {K}_3\varepsilon _3,\ \left\| R^{T}-\widetilde{R}^{T}\right\| \le \mathscr {K}_4\varepsilon _4, \end{aligned}$$

$$\begin{aligned} \left\| I^{RC}-\widetilde{I}^{RC}\right\|&\le \mathscr {K}_5\varepsilon _5,\ \left\| A^{C}-\widetilde{A}^{C}\right\| \le \mathscr {K}_6\varepsilon _6,\ \left\| I^{C}-\widetilde{I}^{C}\right\| \le \mathscr {K}_7\varepsilon _7,\ \left\| R^{C}-\widetilde{R}^{C}\right\| \le \mathscr {K}_8\varepsilon _8, \\ \left\| I^{RT}-\widetilde{I}^{RT}\right\|&\le \mathscr {K}_9\varepsilon _9, \left\| L^{TC}-\widetilde{L}^{TC}\right\| \le \mathscr {K}_{10}\varepsilon _{10},\ \left\| I^{TC}-\widetilde{I}^{TC}\right\| \le \mathscr {K}_{11}\varepsilon _{11},\ \left\| R-\widetilde{R}\right\| \le \mathscr {K}_{12}\varepsilon _{12}. \end{aligned}$$

### Remark 1

Consider that the function $\widetilde{S}^{TC}$ is a solution of the first inequality (27), if a continuous function $h_1$ exists so that

- $$|h_1(s)|< \varepsilon _1$$, and
- $${}^{\mathscr {F}\mathscr {F}\mathscr {M}}_{{\,\,\,\,\,\,\,\,\,\,\,\,0}}{\mathscr {D}}_{s}^{k_1,k_2}S^{TC}(s)={\mathscr {Q}}_{1}(s,S^{TC})+h_1(s)$$.

Similarly, one can indicate such a definition for each of solutions to inequalities (27) by finding $h_i$ for $i\in \mathscr {N}_2^{12}$.

### Theorem 4

Under the assumption $\mathscr {H}$, the model (1) is Ulam-Hyers stable.

### Proof

Let $\varepsilon _1 > 0$ and the function $S^{TC}$ be arbitrary so that

$$\begin{aligned} \begin{aligned} \left| {}^{{\mathscr {F}}{\mathscr {F}}{\mathscr {M}}}_{\,\,\,\,\,\,\,\,\,\,\,\,\text {0}}\mathscr {D}_{s}^{k_1,k_2}S^{TC}(s)-\mathscr {Q}_{1}(s,S^{TC})\right|&\le \varepsilon _1. \end{aligned} \end{aligned}$$

In view of Remark 1, we have a function $h_1$ with $|h_1(s)| < \varepsilon _1$ satisfies

$$\begin{aligned} {}^{{\mathscr {F}}{\mathscr {F}}{\mathscr {M}}}_{\,\,\,\,\,\,\,\,\,\,\,\,\text {0}}\mathscr {D}_{s}^{k_1,k_2}S^{TC}(s)=\mathscr {Q}_{1}(s,S^{TC})+h_1(s). \end{aligned}$$

Consequently,

$$\begin{aligned} \begin{aligned} S^{TC}(s)&=S^{TC}(0)+\frac{k_1k_2}{\mathscr{A}\mathscr{B}(k_1)\Gamma (k_1)}\int _{0}^{s}u^{k_2-1}(s-u)^{k_1-1}\mathscr {Q}_{1}(u,\widetilde{S}^{TC}(u))du+\frac{k_2(1-k_1)s^{k_2-1}}{\mathscr{A}\mathscr{B}(k_1)}\mathscr {Q}_{1}(s,S^{TC}(s))\\&\quad +\frac{k_1k_2}{\mathscr{A}\mathscr{B}(k_1)\Gamma (k_1)}\int _{0}^{s}u^{k_2-1}(s-u)^{k_1-1} h_1(u)du+\frac{k_2(1-k_1)s^{k_2-1}}{\mathscr{A}\mathscr{B}(k_1)}h_{1}(s). \end{aligned} \end{aligned}$$

Let $\widetilde{S}^{TC}$ as the unique solution of the given model, then

$$\begin{aligned} \begin{aligned} \widetilde{S}^{TC}(s)&=\widetilde{S}^{TC}(0)+\frac{k_1k_2}{\mathscr{A}\mathscr{B}(k_1)\Gamma (k_1)}\int _{0}^{s}u^{k_2-1}(s-u)^{k_1-1}\mathscr {Q}_{1}(u,\widetilde{S}^{TC}(u))du+\frac{k_2(1-k_1)s^{k_2-1}}{\mathscr{A}\mathscr{B}(k_1)}\mathscr {Q}_{1}(s,\widetilde{S}^{TC}(s)). \end{aligned} \end{aligned}$$

Hence,

$$\begin{aligned} \Big |S^{TC}(s)-\widetilde{S}^{TC}(s)\Big |&\le \frac{k_1k_2}{\mathscr{A}\mathscr{B}(k_1)\Gamma (k_1)}\int _{0}^{s}u^{k_2-1}(s-u)^{k_1-1}\Big |\mathscr {Q}_{1}(u,S^{TC}(u))-\mathscr {Q}_{1}(u,\widetilde{S}^{TC}(u))\Big |du\nonumber \\&\quad +\frac{k_2(1-k_1)s^{k_2-1}}{\mathscr{A}\mathscr{B}(k_1)}\Big |\mathscr {Q}_{1}(s,S^{TC}(s))-\mathscr {Q}_{1}(s,\widetilde{S}^{TC}(s))\Big |\\&\quad +\frac{k_1k_2}{\mathscr{A}\mathscr{B}(k_1)\Gamma (k_1)}\int _{0}^{s}u^{k_2-1}(s-u)^{k_1-1}\Big |h_{1}(u)\Big |du\nonumber \\&\quad +\frac{k_2(1-k_1)s^{k_2-1}}{\mathscr{A}\mathscr{B}(k_1)}\Big |h_{1}(s)\Big |,\\&\le \left[ \frac{k_1k_2\Gamma (k_2)}{\mathscr{A}\mathscr{B}(k_1)\Gamma (k_1+k_2)}+\frac{k_2(1-k_1)}{\mathscr{A}\mathscr{B}(k_1)}\right] \Psi _1 \Big |S^{TC}-\widetilde{S}^{TC}\Big |\\&\quad +\left[ \frac{k_1k_2\Gamma (k_2)}{\mathscr{A}\mathscr{B}(k_1)\Gamma (k_1+k_2)}+\frac{k_2(1-k_1)}{\mathscr{A}\mathscr{B}(k_1)}\right] \varepsilon _1, \\ \Vert S^{TC}-\widetilde{S}^{TC}\Vert&\le \frac{\left[ \frac{k_1k_2\Gamma (k_2)}{\mathscr{A}\mathscr{B}(k_1)\Gamma (k_1+k_2)}+\frac{k_2(1-k_1)}{\mathscr{A}\mathscr{B}(k_1)}\right] \varepsilon _1}{1-\left[ \frac{k_1k_2\Gamma (k_2)}{\mathscr{A}\mathscr{B}(k_1)\Gamma (k_1+k_2)}+\frac{k_2(1-k_1)}{\mathscr{A}\mathscr{B}(k_1)}\right] \Psi _1}. \end{aligned}$$

Then

$$\begin{aligned} \Vert S^{TC}-\widetilde{S}^{TC}\Vert \le \mathscr {K}_1\varepsilon _1. \end{aligned}$$

here,

$$\begin{aligned} \mathscr {K}_1=\frac{\left[ \frac{k_1k_2\Gamma (k_2)}{\mathscr{A}\mathscr{B}(k_1)\Gamma (k_1+k_2)}+\frac{k_2(1-k_1)}{\mathscr{A}\mathscr{B}(k_1)}\right] }{1-\left[ \frac{k_1k_2\Gamma (k_2)}{\mathscr{A}\mathscr{B}(k_1)\Gamma (k_1+k_2)}+\frac{k_2(1-k_1)}{\mathscr{A}\mathscr{B}(k_1)}\right] \Psi _1}. \end{aligned}$$

Now, applying a similar approach, we have

$$\begin{aligned} \begin{aligned} {\left\{ \begin{array}{ll} \Vert L^{T}-\widetilde{L}^{T}\Vert \le \mathscr {K}_2\varepsilon _2,\\ \Vert I^{T}-\widetilde{I}^{T}\Vert \le \mathscr {K}_3\varepsilon _3,\\ \Vert R^{T}-\widetilde{R}^{T}\Vert \le \mathscr {K}_4\varepsilon _4,\\ \Vert I^{RC}-\widetilde{I}^{RC}\Vert \le \mathscr {K}_5\varepsilon _5,\\ \Vert A^{C}-\widetilde{A}^{C}\Vert \le \mathscr {K}_6\varepsilon _6,\\ \Vert I^{C}-\widetilde{I}^{C}\Vert \le \mathscr {K}_7\varepsilon _7,\\ \Vert R^{C}-\widetilde{R}^{C}\Vert \le \mathscr {K}_8\varepsilon _8,\\ \Vert I^{RT}-\widetilde{I}^{RT}\Vert \le \mathscr {K}_9\varepsilon _9,\\ \Vert L^{TC}-\widetilde{L}^{TC}\Vert \le \mathscr {K}_{10}\varepsilon _{10},\\ \Vert I^{TC}-\widetilde{I}^{TC}\Vert \le \mathscr {K}_{11}\varepsilon _{11}\\ \Vert R-\widetilde{R}\Vert \le \mathscr {K}_{12}\varepsilon _{12}. \end{array}\right. } \end{aligned} \end{aligned}$$

Hence, we conclude the fractal-fractional model (1) is Ulam-Hyers stable. This completes the proof. $\square$

## Numerical scheme

In this segment, the numerical scheme are analyzes for the proposed model (1). For the numerical scheme, we consider the equation of the Atangana-Baleanu fractional operator can also as follows:

$$\begin{aligned} {}^{{\mathscr {F}}{\mathscr {F}}{\mathscr {M}}}_{\,\,\,\,\,\,\,\,\,\,\,\,\text {0}}\mathscr {D}_{s}^{k_1,k_2}y(s)=k_2 s^{k_2-1}\mathscr {Q}(s,y(s)). \end{aligned}$$

Utilizing the fractal-fractional integral operator having generalized Mittag-Leffler type kernel, we obtain

$$\begin{aligned} y(s)=y(0)+\frac{k_2(1-k_1)s^{k_2-1}}{\mathscr{A}\mathscr{B}(k_1)}\mathscr {Q}(s,y(s))+\frac{k_1k_2}{\mathscr{A}\mathscr{B}(k_1)\Gamma (k_1)}\int _{0}^{s}u^{k_2-1}(s-u)^{k_1-1} \mathscr {Q}(u,y(u))du. \end{aligned}$$

**Figure 2:**
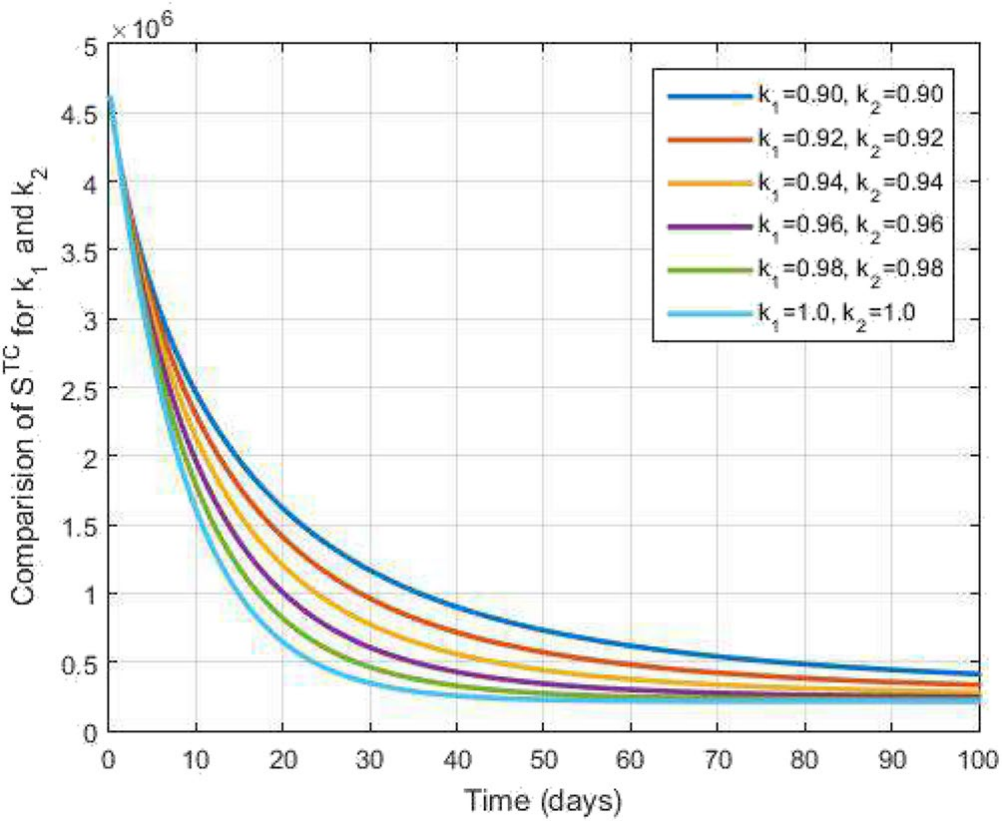
Susceptible to both TB and COVID-19 and time variations for varying values of $k_1$ and $k_2$.

**Figure 3:**
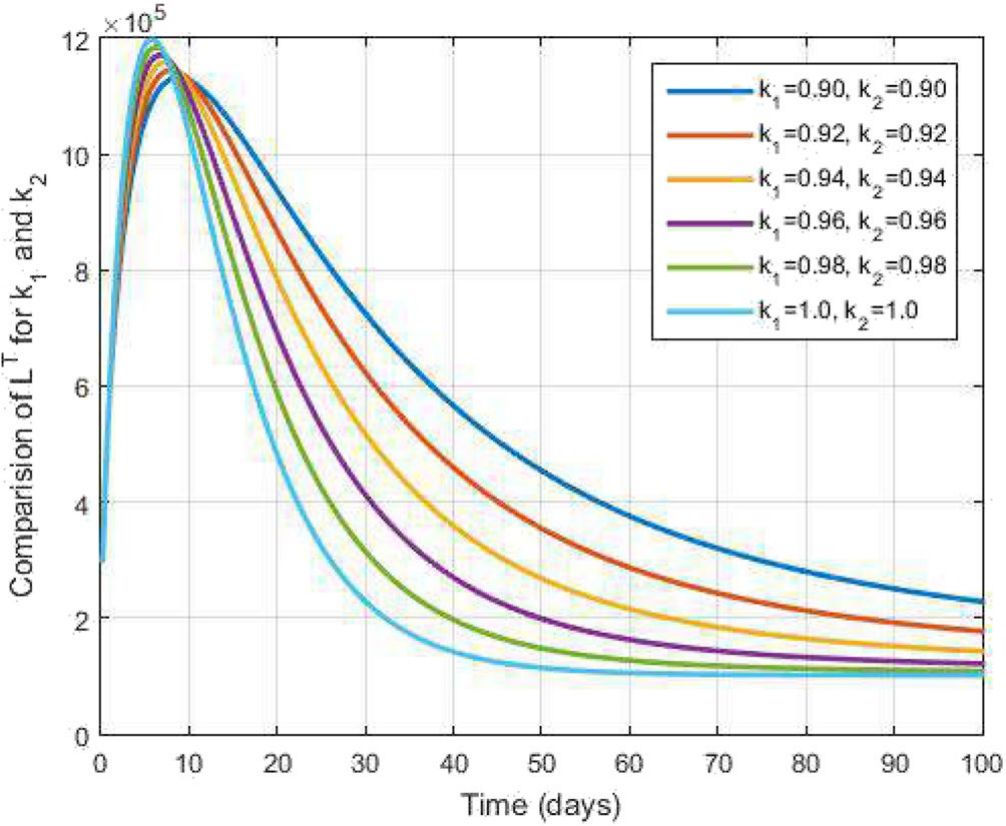
Latent level TB infected people and time variations for varying values of $k_1$ and $k_2$.

Now, at $s=s_{n+1}$, which gives

39 $$\begin{aligned} \begin{aligned} y_{n+1}&=y(0)+\frac{k_2(1-k_1)s_n^{k_2-1}}{\mathscr{A}\mathscr{B}(k_1)}\mathscr {Q}(s_n,y(s_n))+\frac{k_1k_2}{\mathscr{A}\mathscr{B}(k_1)\Gamma (k_1)}\int _{0}^{s}u^{k_2-1}(s_{n+1}-u)^{k_1-1} \mathscr {Q}(u,y(u))du, \end{aligned} \end{aligned}$$

which can be written as

$$\begin{aligned} \begin{aligned} y_{n+1}&=y(0)+\frac{(1-k_1)}{\mathscr{A}\mathscr{B}(k_1)}\mathscr {U}(s_n,y(s_n))+\frac{k_1k_2}{\mathscr{A}\mathscr{B}(k_1)\Gamma (k_1)}\sum _{\gamma =1}^{n}\Bigg [\frac{\mathscr {U}(s_{\gamma },y(s_{\gamma }))}{h}\int _{s_{\gamma }}^{s_{\gamma +1}}(u-s_{\gamma -1})(s_{n+1}-u)^{k_1-1}\ du\\&\quad -\frac{\mathscr {U}(s_{\gamma -1},y(s_{\gamma -1}))}{h}\int _{s_{\gamma }}^{s_{\gamma +1}}(u-s_{\gamma })(s_{n+1}-u)^{k_1-1}\ du\Bigg ]. \end{aligned} \end{aligned}$$

**Figure 4:**
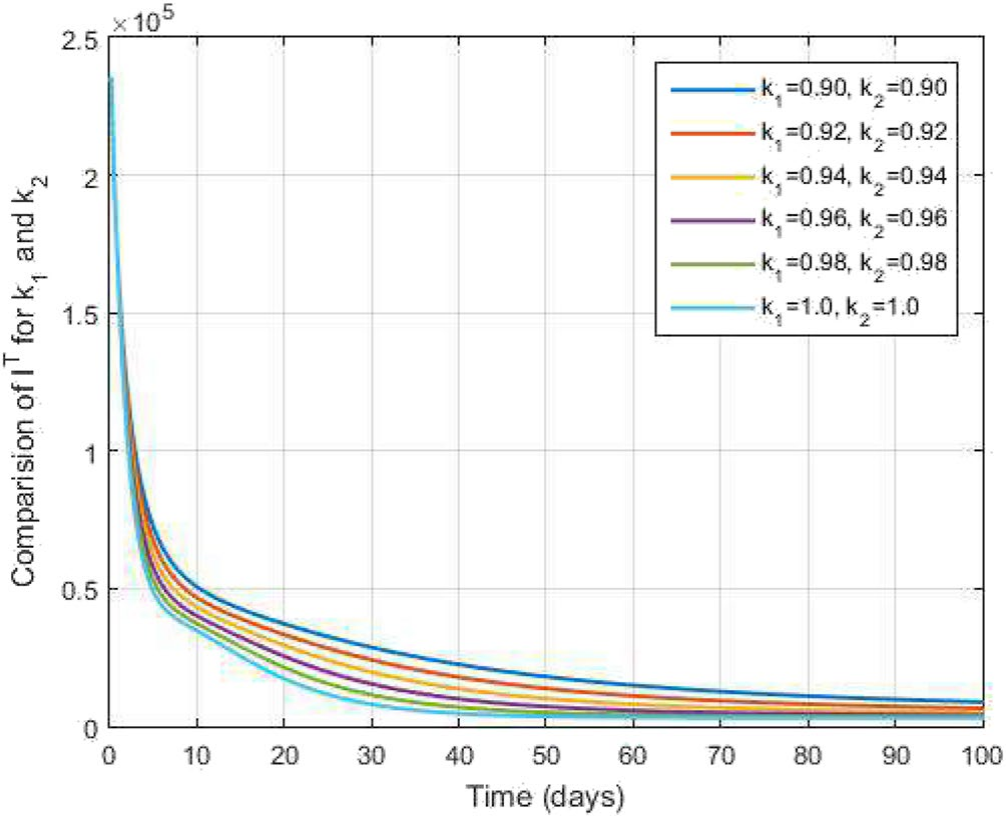
Active level TB infected people and time variations for varying values of $k_1$ and $k_2$.

**Figure 5:**
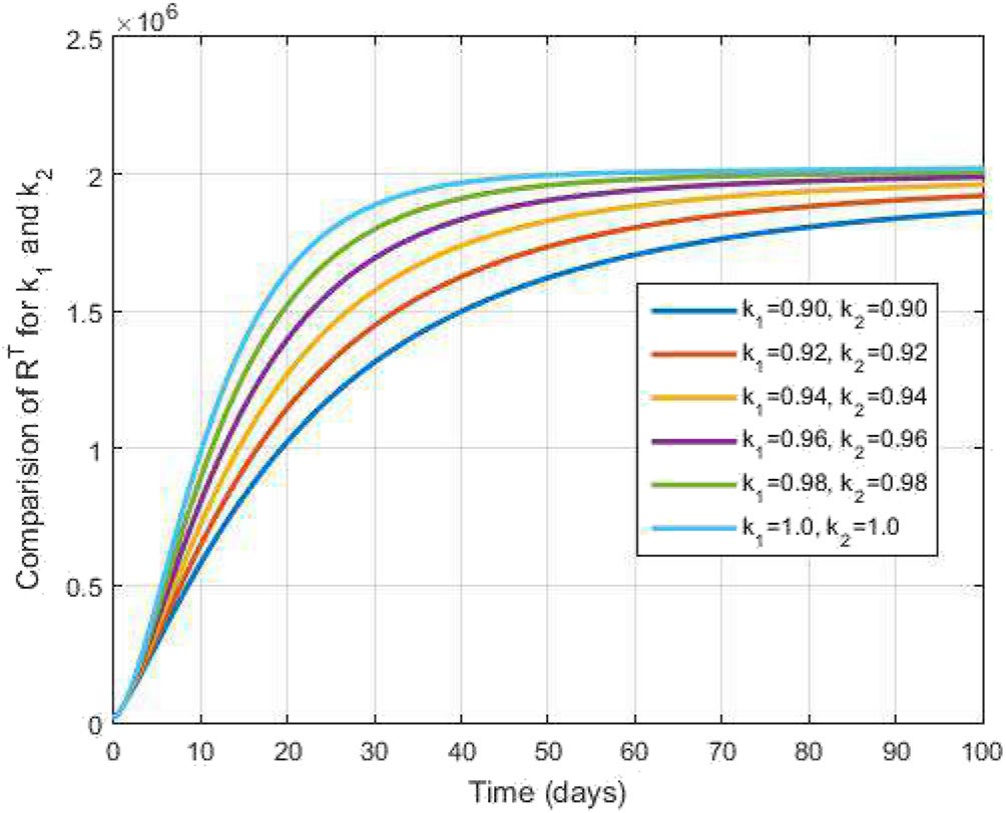
Recovered from TB infected people and time variations for varying values of $k_1$ and $k_2$.

Utilizing the Lagrange polynomial interpolation to Eq. (39), we obtain

$$\begin{aligned} \begin{aligned} y_{n+1}&=y(0)+k_2s_n^{k_2-1}\frac{1-k_1}{\mathscr{A}\mathscr{B}(k_1)}\mathscr {U}(s_n,y(s_n))+\frac{k_1h^{k_1}}{\mathscr{A}\mathscr{B}(k_1)\Gamma (k_1+2)}\sum _{\gamma =1}^{n}\Bigg [\mathscr {U}(s_{\gamma },y(s_{\gamma }))\\&\qquad \times \Big ((n+1-\gamma )^{k_1}(n-\gamma +2+k_1)-(n-\gamma )^{k_1}(n-\gamma +2+2\ k_1)\Big )\\ {}&\quad -\mathscr {U}(s_{\gamma -1},y(s_{\gamma -1}))\Big ((n+1-\gamma )^{k_1+1}-(n-\gamma +1+k_1)(n-\gamma )^{k_1}\Big )\Bigg ]. \end{aligned} \end{aligned}$$

For clarity, we can write the as follows:

$$\begin{aligned} \begin{aligned} y_{n+1}&=y(0)+k_2s_n^{k_2-1}\frac{1-k_1}{\mathscr{A}\mathscr{B}(k_1)}\mathscr {Q}(s_n,y(s_n))+\frac{k_2h^{k_1}}{\mathscr{A}\mathscr{B}(k_1)\Gamma (k_1+2)}\sum _{\gamma =1}^{n}\Bigg [s_{\gamma }^{k_2-1}\mathscr {Q}(s_{\gamma },y(s_{\gamma }))\\&\quad \times \Big ((n+1-\gamma )^{k_1}(n-\gamma +2+k_1)-(n-\gamma )^{k_1}(n-\gamma +2+2\ k_1)\Big )\\ {}&\quad -s_{\gamma -1}^{k_2-1}\mathscr {Q}(s_{\gamma -1},y(s_{\gamma -1}))\Big ((n+1-\gamma )^{k_1+1}-(n-\gamma +1+k_1)(n-\gamma )^{k_1}\Big )\Bigg ]. \end{aligned} \end{aligned}$$

**Figure 6:**
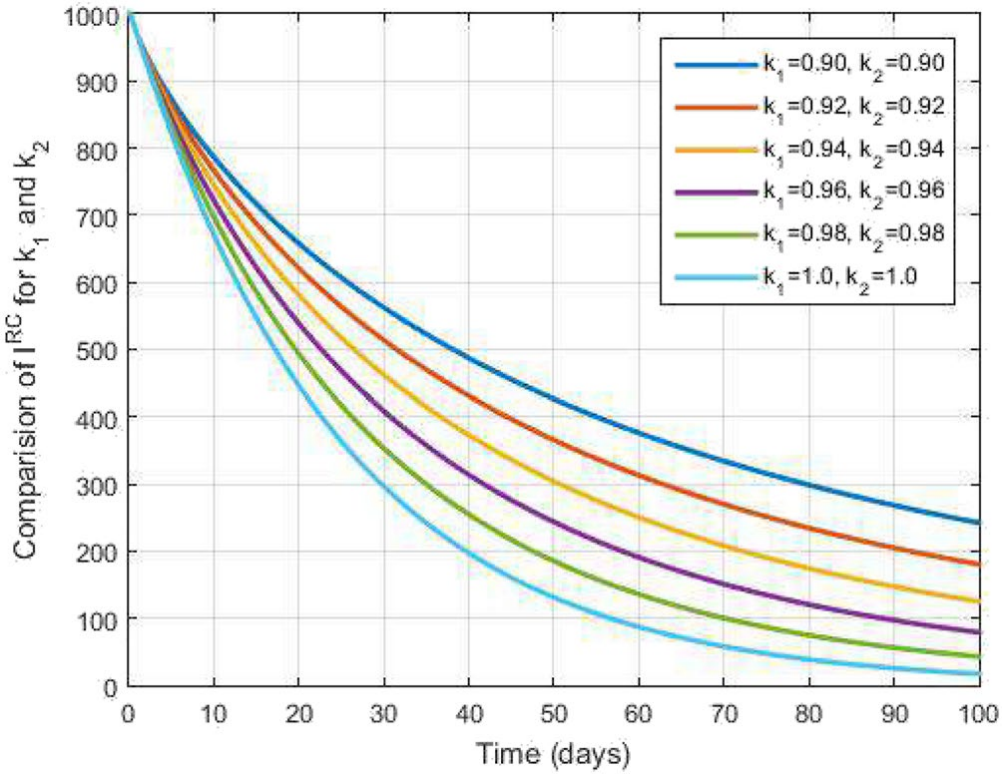
Infected with COVID-19 after recovering from TB people and time variations for varying values of $k_1$ and $k_2$.

**Figure 7:**
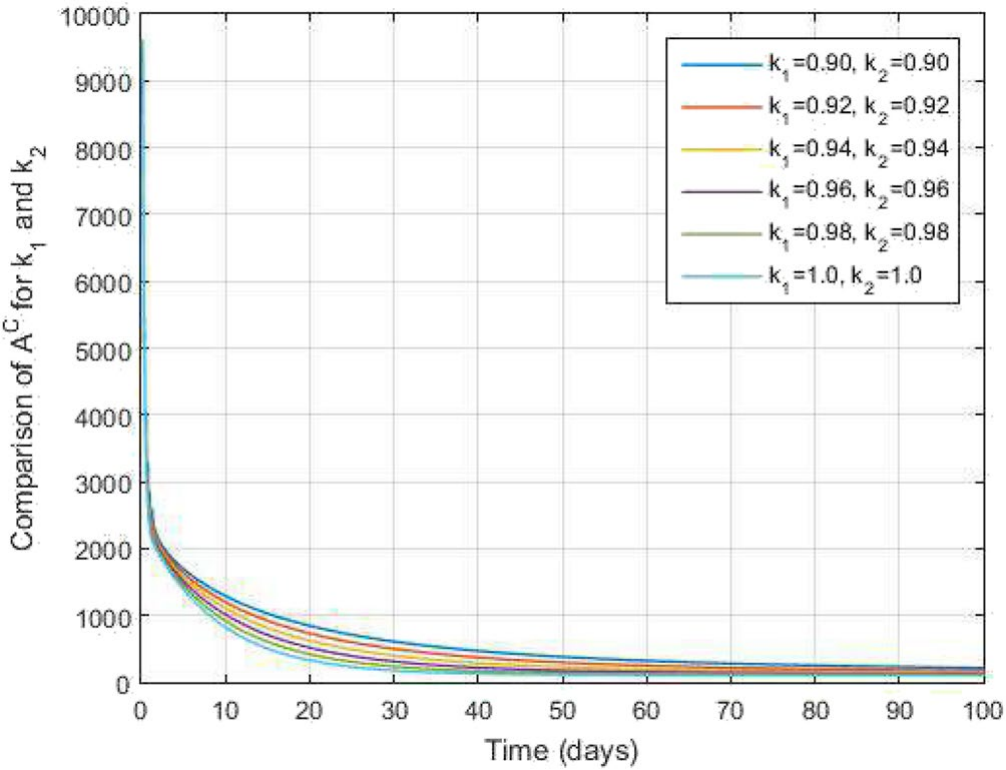
Asymptomatic COVID-19 infected people and time variations for varying values of $k_1$ and $k_2$.

**Figure 8:**
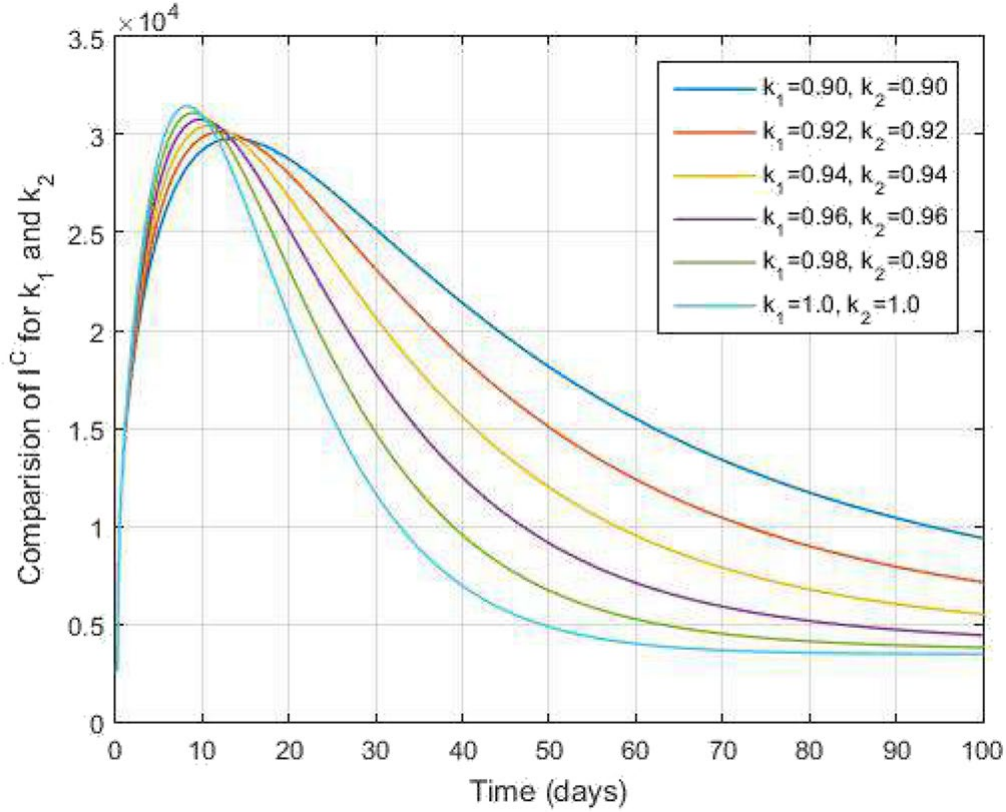
Symptomatic COVID-19 infected people and time variations for varying values of $k_1$ and $k_2$.

**Figure 9:**
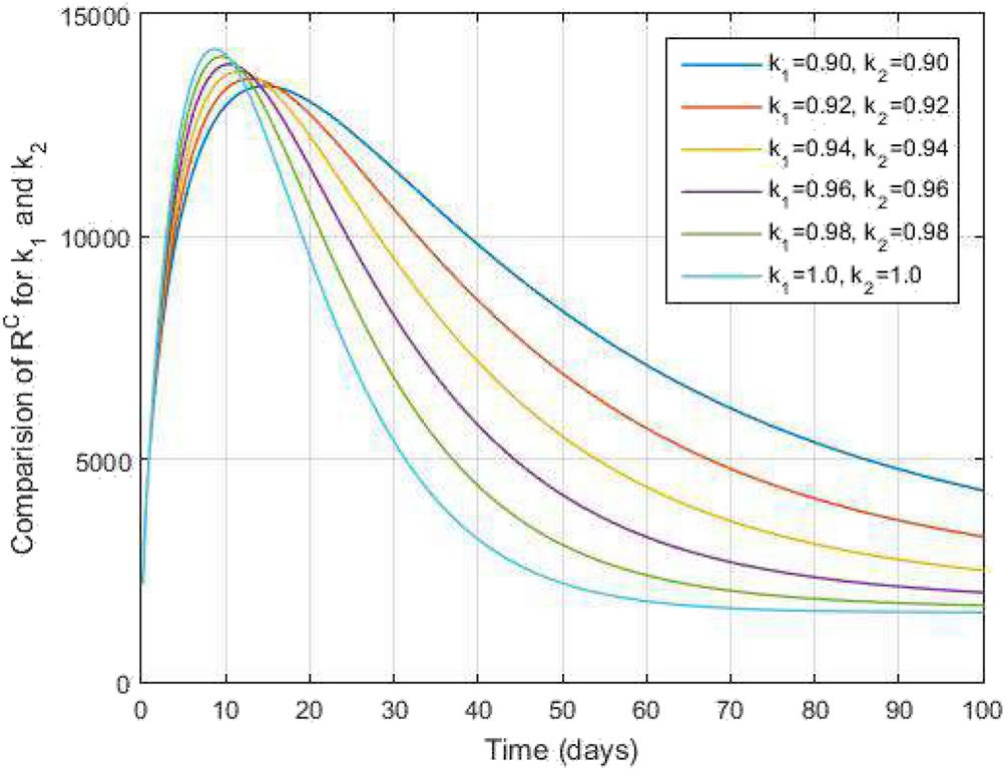
Recovered from COVID-19 infected people and time variations for varying values of $k_1$ and $k_2$.

Thus, by assuming

$$\begin{aligned} \begin{aligned} y_1(n,\gamma )&:=(n+1-\gamma )^{k_1}(n-\gamma +2+k_1)-(n-\gamma )^{k_1}(n-\gamma +2+2\ k_1),\\ y_2(n,\gamma )&:=(n+1-\gamma )^{k_1+1}-(n-\gamma +1+k_1)(n-\gamma )^{k_1}, \end{aligned} \end{aligned}$$

the numerical scheme for the integral system Eqs. (2) to (13) is obtained as

$$\begin{aligned} \begin{aligned} S^{TC}(s_{n+1})&=S^{TC}(0)+k_2s_n^{k_2-1}\frac{1-k_1}{\mathscr{A}\mathscr{B}(k_1)}\mathscr {Q}_1(s_n,S^{TC}(s_n))+\frac{k_2h^{k_1}}{\mathscr{A}\mathscr{B}(k_1)\Gamma (k_1+2)}\\&\quad \times \sum _{\gamma =1}^{n}\Bigg [s_{\gamma }^{k_2-1}\mathscr {Q}_1(s_{\gamma },S^{TC}(s_{\gamma }))y_1(n,\gamma )-s_{\gamma -1}^{k_2-1}\mathscr {Q}_1(s_{\gamma -1},S^{TC}(s_{\gamma -1}))y_2(n,\gamma )\Bigg ]. \end{aligned} \end{aligned}$$

Similarly, the rest of the compartments $L^T$, $I^T$, $R^T$, $I^{RC}$, $A^C$, $I^C$, $R^C$, $I^{RT}$, $L^{TC}$, $I^{TC}$ and *R* we calculate the same numerical scheme as follows:

$$\begin{aligned} L^{T}(s_{n+1})&=L^{T}(0)+k_2s_n^{k_2-1}\frac{1-k_1}{\mathscr{A}\mathscr{B}(k_1)}\mathscr {Q}_2(s_n,L^{T}(s_n))+\frac{k_2h^{k_1}}{\mathscr{A}\mathscr{B}(k_1)\Gamma (k_1+2)}\\&\quad \times \sum _{\gamma =1}^{n}\Bigg [s_{\gamma }^{k_2-1}\mathscr {Q}_2(s_{\gamma },L^{T}(s_{\gamma }))y_1(n,\gamma )-s_{\gamma -1}^{k_2-1}\mathscr {Q}_2(s_{\gamma -1},L^{T}(s_{\gamma -1}))y_2(n,\gamma )\Bigg ], \\ I^{T}(s_{n+1})&=I^{T}(0)+k_2s_n^{k_2-1}\frac{1-k_1}{\mathscr{A}\mathscr{B}(k_1)}\mathscr {Q}_3(s_n,I^{T}(s_n))+\frac{k_2h^{k_1}}{\mathscr{A}\mathscr{B}(k_1)\Gamma (k_1+2)}\\&\quad \times \sum _{\gamma =1}^{n}\Bigg [s_{\gamma }^{k_2-1}\mathscr {Q}_3(s_{\gamma },I^{T}(s_{\gamma }))y_1(n,\gamma )-s_{\gamma -1}^{k_2-1}\mathscr {Q}_3(s_{\gamma -1},I^{T}(s_{\gamma -1}))y_2(n,\gamma )\Bigg ], \\ R^{T}(s_{n+1})&=R^{T}(0)+k_2s_n^{k_2-1}\frac{1-k_1}{\mathscr{A}\mathscr{B}(k_1)}\mathscr {Q}_4(s_n,R^{T}(s_n))+\frac{k_2h^{k_1}}{\mathscr{A}\mathscr{B}(k_1)\Gamma (k_1+2)}\\&\quad \times \sum _{\gamma =1}^{n}\Bigg [s_{\gamma }^{k_2-1}\mathscr {Q}_4(s_{\gamma },R^{T}(s_{\gamma }))y_1(n,\gamma )-s_{\gamma -1}^{k_2-1}\mathscr {Q}_4(s_{\gamma -1},R^{T}(s_{\gamma -1}))y_2(n,\gamma )\Bigg ], \end{aligned}$$

**Figure 10:**
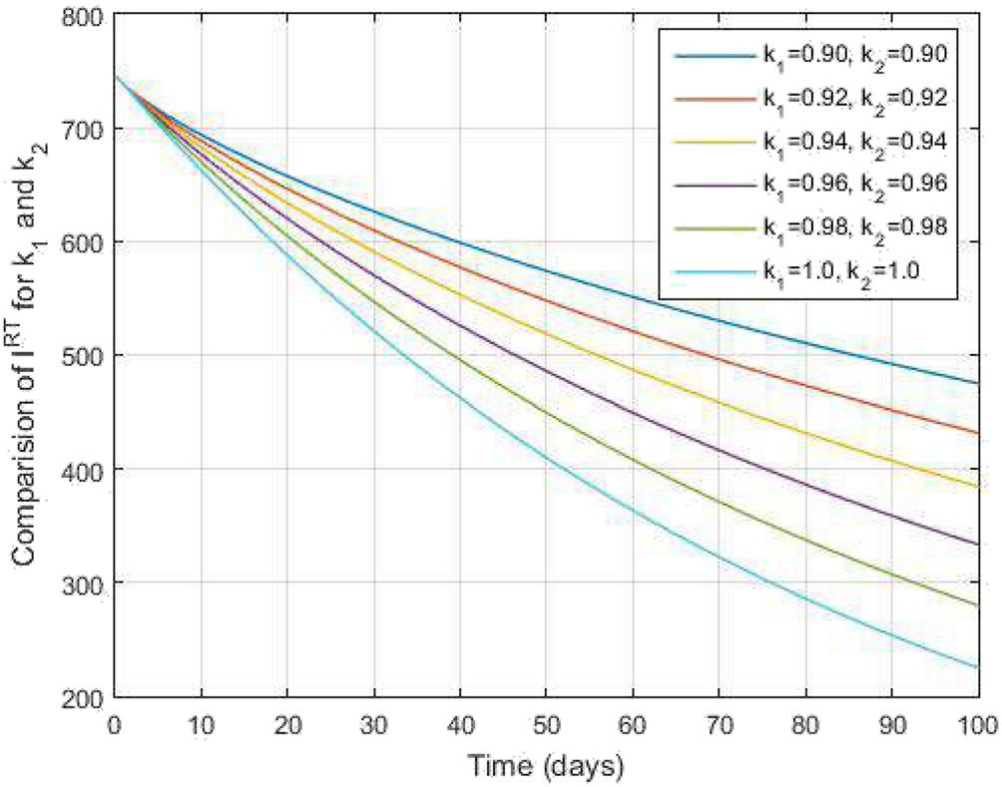
Infected with TB after recovering from COVID-19 people and time variations for varying values of $k_1$ and $k_2$.

**Figure 11:**
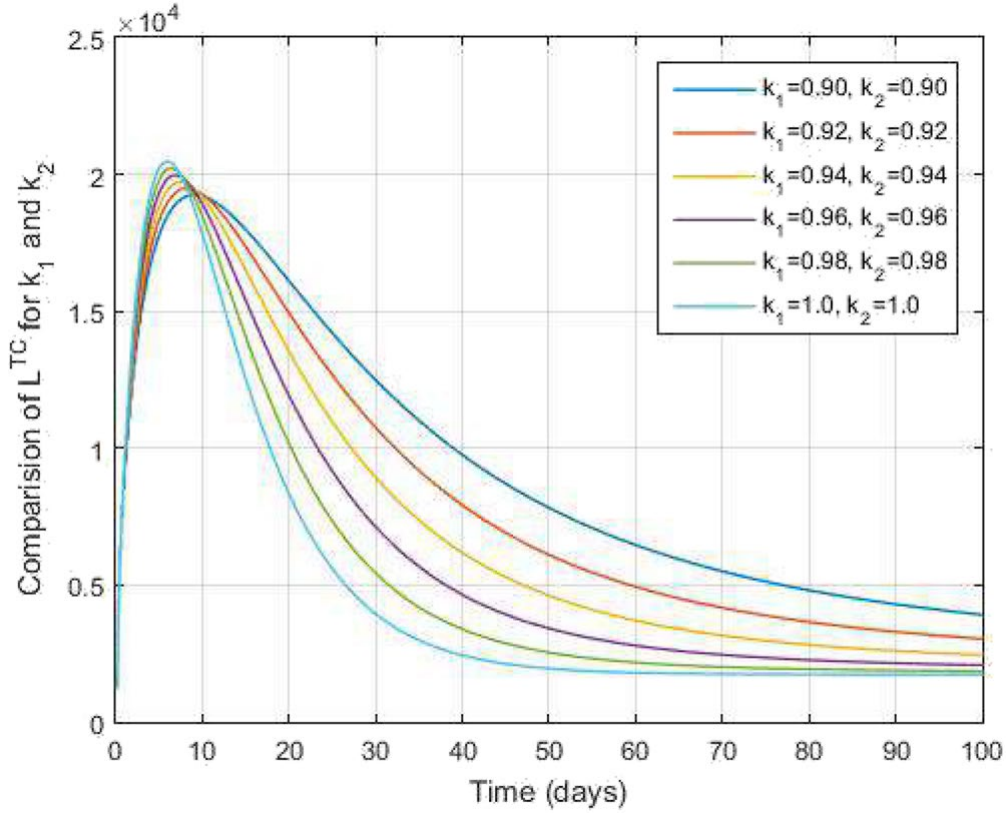
Both latent TB and COVID-19 co-infected people and time variations for varying values of $k_1$ and $k_2$.

$$\begin{aligned} I^{RC}(s_{n+1})&=I^{RC}(0)+k_2s_n^{k_2-1}\frac{1-k_1}{\mathscr{A}\mathscr{B}(k_1)}\mathscr {Q}_5(s_n,I^{RC}(s_n))+\frac{k_2h^{k_1}}{\mathscr{A}\mathscr{B}(k_1)\Gamma (k_1+2)}\\&\quad \times \sum _{\gamma =1}^{n}\Bigg [s_{\gamma }^{k_2-1}\mathscr {Q}_5(s_{\gamma },I^{RC}(s_{\gamma }))y_1(n,\gamma )-s_{\gamma -1}^{k_2-1}\mathscr {Q}_5(s_{\gamma -1},I^{RC}(s_{\gamma -1}))y_2(n,\gamma )\Bigg ], \\ A^{C}(s_{n+1})&=A^{C}(0)+k_2s_n^{k_2-1}\frac{1-k_1}{\mathscr{A}\mathscr{B}(k_1)}\mathscr {Q}_6(s_n,A^{C}(s_n))+\frac{k_2h^{k_1}}{\mathscr{A}\mathscr{B}(k_1)\Gamma (k_1+2)}\\&\quad \times \sum _{\gamma =1}^{n}\Bigg [s_{\gamma }^{k_2-1}\mathscr {Q}_6(s_{\gamma },A^{C}(s_{\gamma }))y_1(n,\gamma )-s_{\gamma -1}^{k_2-1}\mathscr {Q}_6(s_{\gamma -1},A^{C}(s_{\gamma -1}))y_2(n,\gamma )\Bigg ], \end{aligned}$$

**Figure 12:**
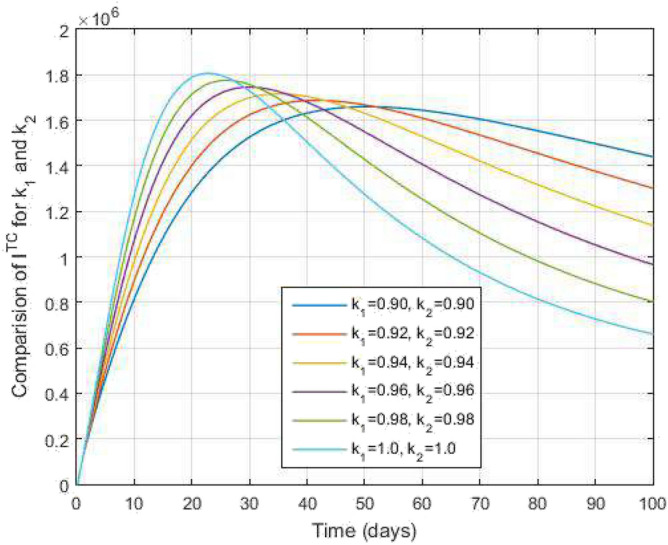
Both active TB and COVID-19 co-infected people and time variations for varying values of $k_1$ and $k_2$.

**Figure 13:**
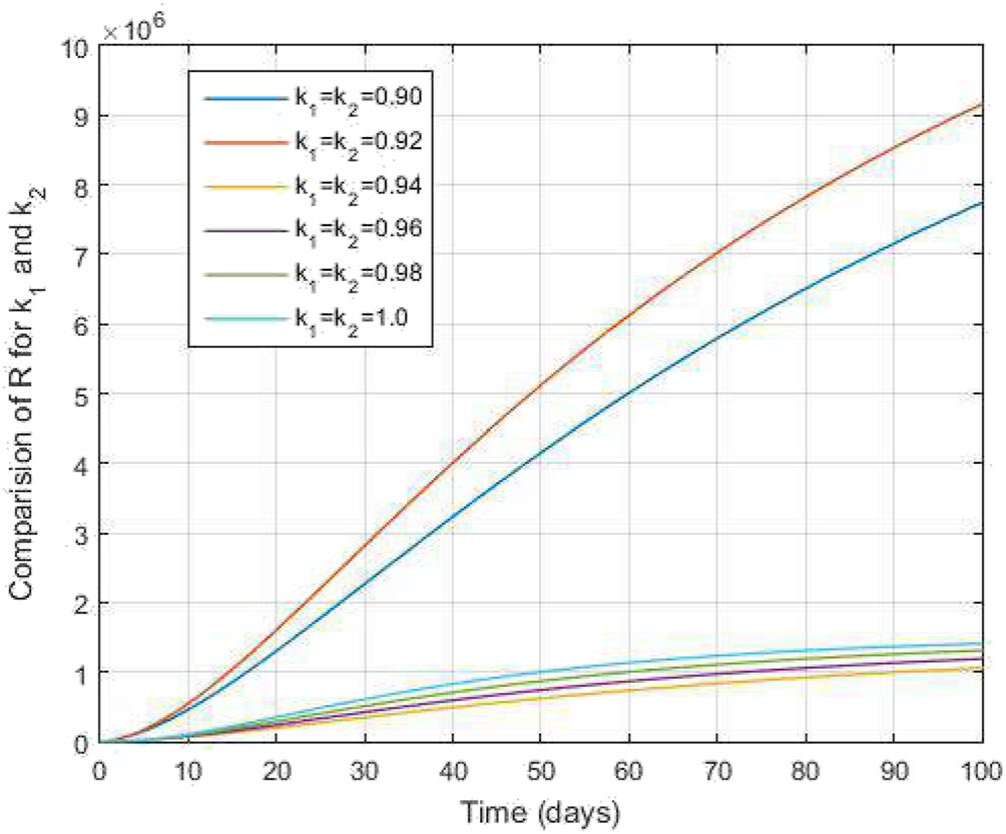
Both TB and COVID-19 recovered people and time variations for varying values of $k_1$ and $k_2$.

$$\begin{aligned} I^{C}(s_{n+1})&=I^{C}(0)+k_2s_n^{k_2-1}\frac{1-k_1}{\mathscr{A}\mathscr{B}(k_1)}\mathscr {Q}_7(s_n,I^{C}(s_n))+\frac{k_2h^{k_1}}{\mathscr{A}\mathscr{B}(k_1)\Gamma (k_1+2)}\\&\quad \times \sum _{\gamma =1}^{n}\Bigg [s_{\gamma }^{k_2-1}\mathscr {Q}_7(s_{\gamma },I^{C}(s_{\gamma }))y_1(n,\gamma )-s_{\gamma -1}^{k_2-1}\mathscr {Q}_7(s_{\gamma -1},I^{C}(s_{\gamma -1}))y_2(n,\gamma )\Bigg ], \\ R^{C}(s_{n+1})&=R^{C}(0)+k_2s_n^{k_2-1}\frac{1-k_1}{\mathscr{A}\mathscr{B}(k_1)}\mathscr {Q}_8(s_n,R^{C}(s_n))+\frac{k_2h^{k_1}}{\mathscr{A}\mathscr{B}(k_1)\Gamma (k_1+2)}\\&\quad \times \sum _{\gamma =1}^{n}\Bigg [s_{\gamma }^{k_2-1}\mathscr {Q}_8(s_{\gamma },R^{C}(s_{\gamma }))y_1(n,\gamma )-s_{\gamma -1}^{k_2-1}\mathscr {Q}_8(s_{\gamma -1},R^{C}(s_{\gamma -1}))y_2(n,\gamma )\Bigg ], \\ I^{RT}(s_{n+1})&=I^{RT}(0)+k_2s_n^{k_2-1}\frac{1-k_1}{\mathscr{A}\mathscr{B}(k_1)}\mathscr {Q}_9(s_n,I^{RT}(s_n))+\frac{k_2h^{k_1}}{\mathscr{A}\mathscr{B}(k_1)\Gamma (k_1+2)}\\&\quad \times \sum _{\gamma =1}^{n}\Bigg [s_{\gamma }^{k_2-1}\mathscr {Q}_9(s_{\gamma },I^{RT}(s_{\gamma }))y_1(n,\gamma )-s_{\gamma -1}^{k_2-1}\mathscr {Q}_9(s_{\gamma -1},I^{RT}(s_{\gamma -1}))y_2(n,\gamma )\Bigg ], \end{aligned}$$

**Figure 14:**
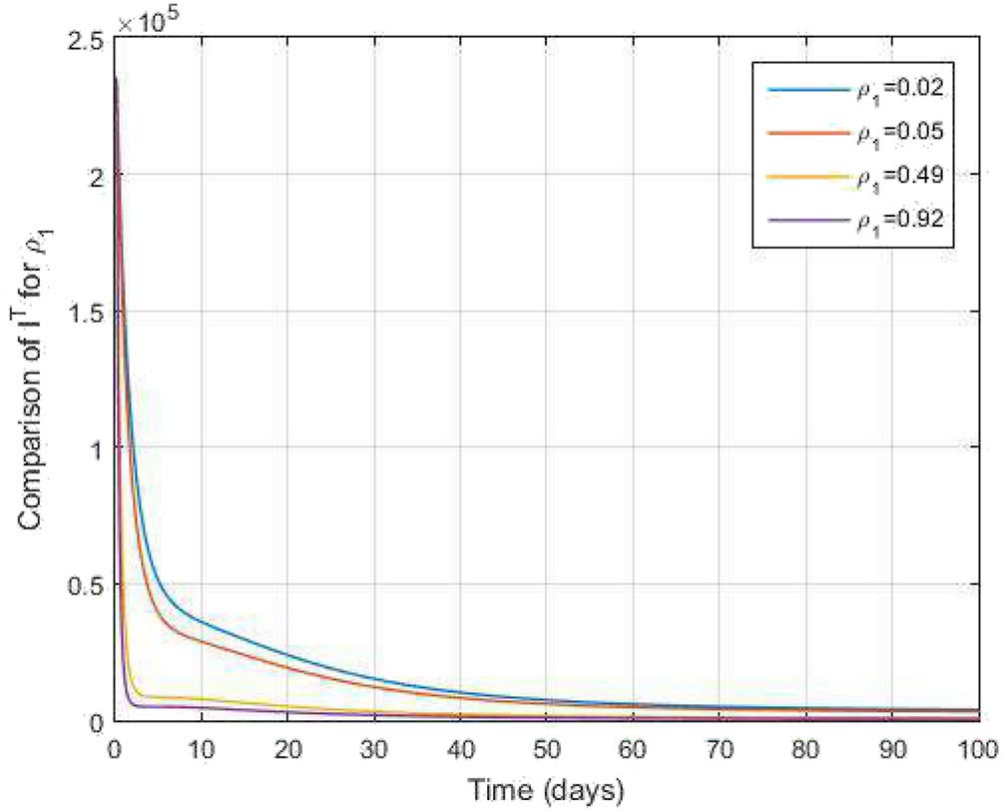
Comparative study of $I^T$ and time variations with $k_1=k_2=0.95$ for varying values of infection rate $\rho _1$.

**Figure 15:**
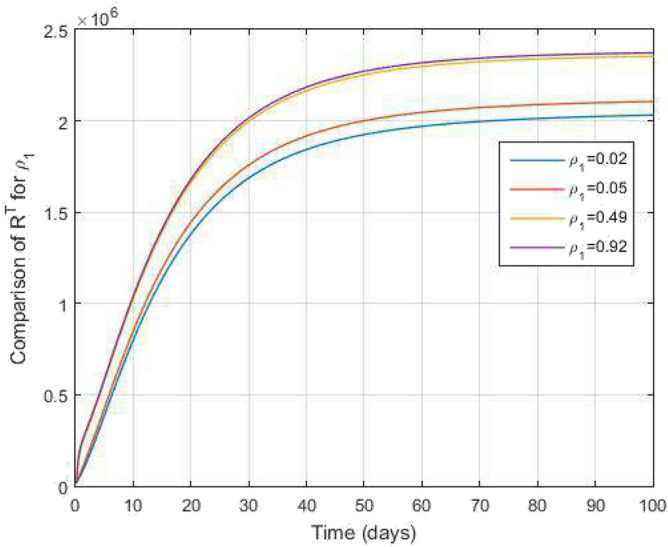
Comparative study of $R^T$ and time variations with $k_1=k_2=0.95$ for varying values of infection rate $\rho _1$.

**Figure 16:**
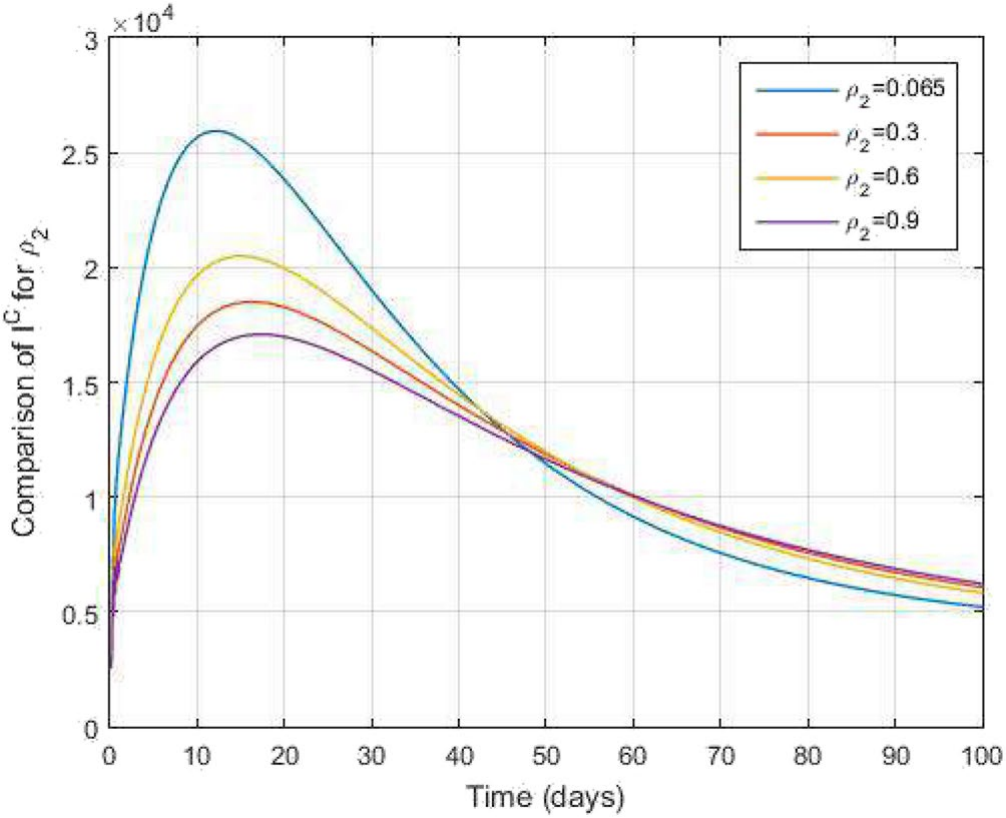
Comparative study of $I^c$ and time variations with $k_1=k_2=0.95$ for varying values of infection rate $\rho _2$.

**Figure 17:**
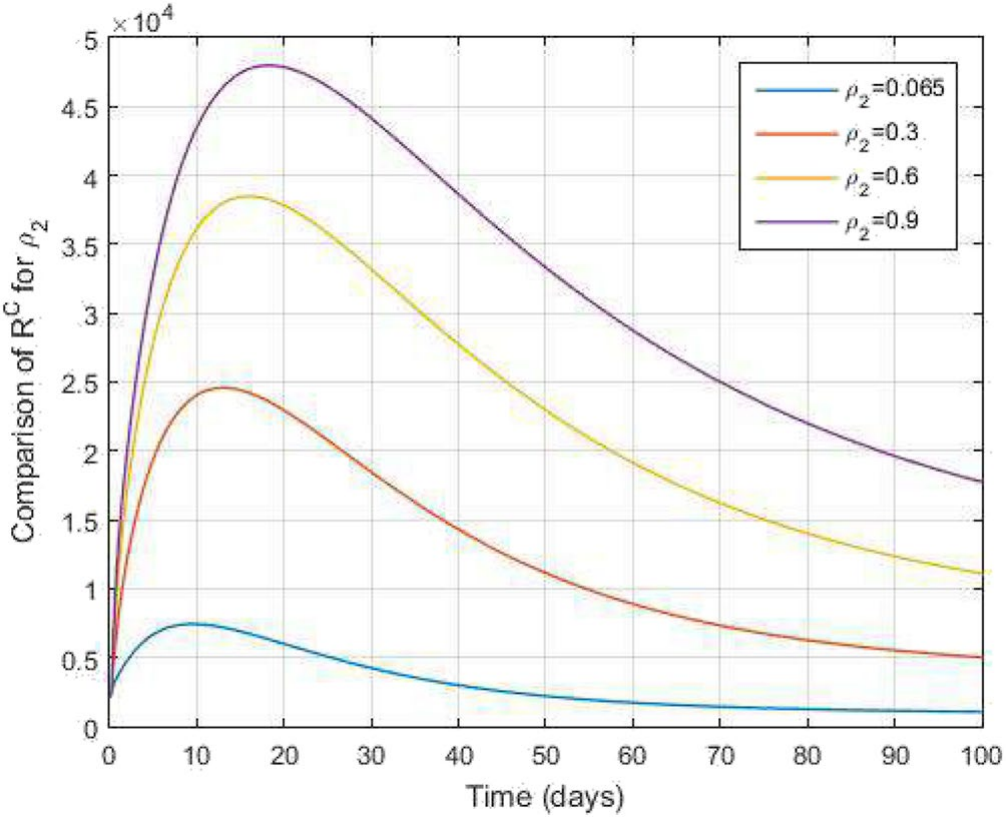
Comparative study of $R^c$ and time variations with $k_1=k_2=0.95$ for varying values of infection rate $\rho _2$.

$$\begin{aligned} L^{TC}(s_{n+1})&=L^{TC}(0)+k_2s_n^{k_2-1}\frac{1-k_1}{\mathscr{A}\mathscr{B}(k_1)}\mathscr {Q}_{10}(s_n,L^{TC}(s_n))+\frac{k_2h^{k_1}}{\mathscr{A}\mathscr{B}(k_1)\Gamma (k_1+2)}\\&\quad \times \sum _{\gamma =1}^{n}\Bigg [s_{\gamma }^{k_2-1}\mathscr {Q}_{10}(s_{\gamma },L^{TC}(s_{\gamma }))y_1(n,\gamma )-s_{\gamma -1}^{k_2-1}\mathscr {Q}_{10}(s_{\gamma -1},L^{TC}(s_{\gamma -1}))y_2(n,\gamma )\Bigg ], \\ I^{TC}(s_{n+1})&=I^{TC}(0)+k_2s_n^{k_2-1}\frac{1-k_1}{\mathscr{A}\mathscr{B}(k_1)}\mathscr {Q}_{11}(s_n,I^{TC}(s_n))+\frac{k_2h^{k_1}}{\mathscr{A}\mathscr{B}(k_1)\Gamma (k_1+2)}\\&\quad \times \sum _{\gamma =1}^{n}\Bigg [s_{\gamma }^{k_2-1}\mathscr {Q}_{11}(s_{\gamma },I^{TC}(s_{\gamma }))y_1(n,\gamma )-s_{\gamma -1}^{k_2-1}\mathscr {Q}_{11}(s_{\gamma -1},I^{TC}(s_{\gamma -1}))y_2(n,\gamma )\Bigg ], \\ R(s_{n+1})&=R(0)+k_2s_n^{k_2-1}\frac{1-k_1}{\mathscr{A}\mathscr{B}(k_1)}\mathscr {Q}_{12}(s_n,R(s_n))+\frac{k_2h^{k_1}}{\mathscr{A}\mathscr{B}(k_1)\Gamma (k_1+2)}\\&\quad \times \sum _{\gamma =1}^{n}\Bigg [s_{\gamma }^{k_2-1}\mathscr {Q}_{12}(s_{\gamma },R(s_{\gamma }))y_1(n,\gamma )-s_{\gamma -1}^{k_2-1}\mathscr {Q}_{12}(s_{\gamma -1},L^{T}(s_{\gamma -1}))y_2(n,\gamma )\Bigg ]. \end{aligned}$$

## Numerical simulation

In this segment, the numerical simulation are examines for the proposed model (1). For this model, the initial values are assumed to be $S^{TC}=4605410,\ L^{T}=300000,\ I^{T}=235000,\ R{T}=20000,\ I^{RC}=1000,\ A^{C}=9600,\ I^{C}=2600,\ R^{C}=2200,\ I^{RT}=745,\ L^{TC}=1250,\ I^{TC}=600$ and $R=125$.

Moreover, we utilized the many parametric values available from^17,18^ and also assumed other parametric values. The parametric values are given by $\pi =6193,\ \lambda _1=0.6,\ \lambda _2=0.659,\ \alpha _1=0.0039,\ \alpha _2=1.1148,\ \alpha _{12}=1.60,\ \eta =1.01,\ \epsilon =1.06,\ \beta _1=0,\ \beta _2=0,\ \omega 1=0.0244,\ \omega 2=0.20,\ \sigma =0.01,\ \tau =0.0039,\ \rho _1=0.0031,\ \rho _2=0.1393,\ r=0.32, \ \mu =0.0012,\ d^T=0$ and $\nu _2=0$.

**Figure 18:**
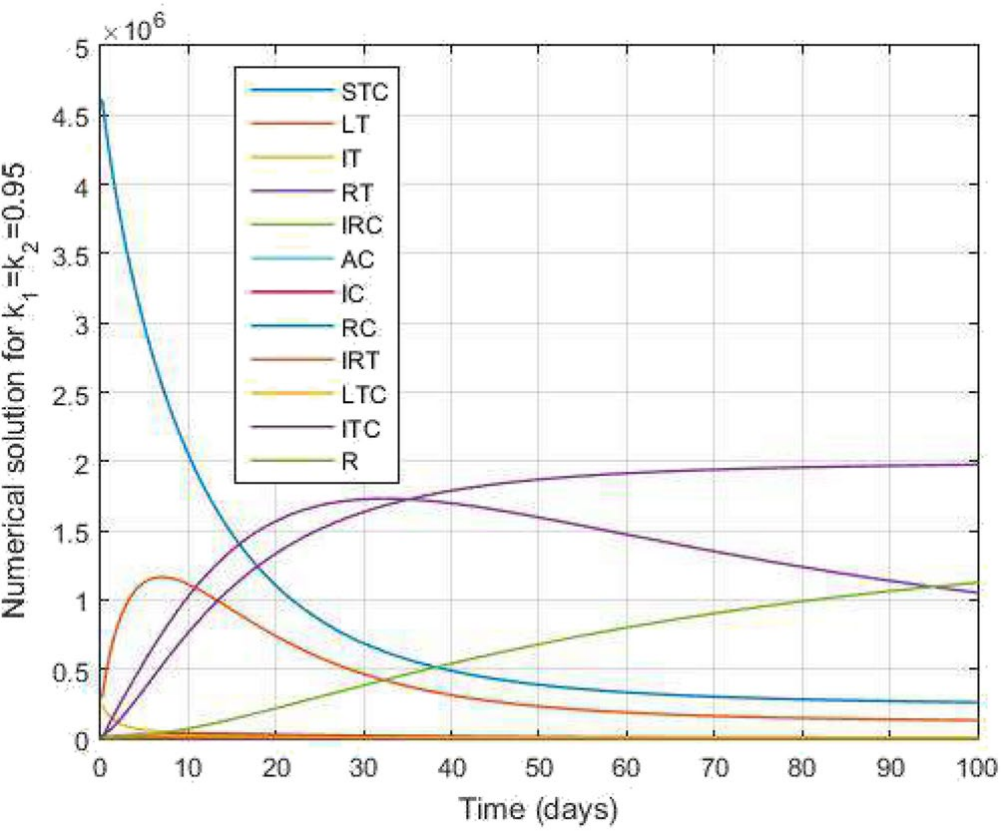
Comparative study of TB and COVID-19 co-infection population density and $k_1=k_2=0.95$ against time.

Figure 2 plots the graphs for TB and COVID-19 susceptible individuals against time and varying $k_1$ and $k_2$. This graph shows that the different values of $k_1$ and $k_2$ in favour of $S^{TC}$ human individuals decrease rapidly and it comes to nearly zero in the long run. In Figs. 3 and 8, the graphs are plotted for the latent TB human population and symptomatic infected from COVID-19 against time and varying $k_1$ and $k_2$. We have found that the corresponding to changing $k_1$ and $k_2$ in favour of $L^{T}$ and $I^{T}$ human individuals decreases to nearly zero. Figures 4 and 6 shows the repercussion of active TB infectious individuals and COVID-19 infected after recovery from TB human individuals. The $I^{T}$ and $I^{RC}$ human individuals decreased by enhancing the $k_1$ and $k_2$ value with time, it comes to nearly zero. During this time, the recovered TB and COVID-19 also reached their maximum peak value concerning $k_1$ and $k_2$, as shown in Figs. 5 and 9. Then the TB and COVID-19 recovered individuals rapidly increased against time with varying values of $k_1$ and $k_2$. In Figs. 7 and 10, the graph represents the symptomatic infectious individuals from COVID-19 and infected with TB after recovery from COVID-19 human individuals decrease rapidly with different values of $k_1$ and $k_2$ in the favouring and in the long run, it comes to nearly zero.

**Figure 19:**
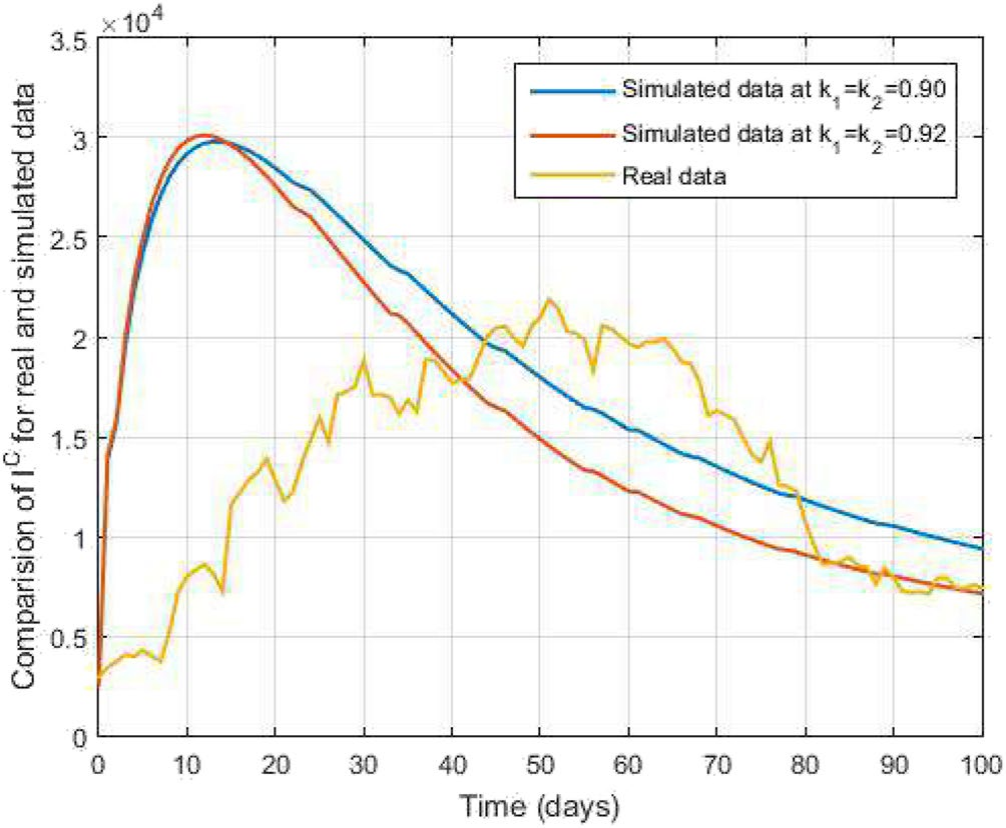
Comparative study of simulated and real data in COVID-19 infected people and time variations for varying of $k_1$ and $k_2$.

In Figs. 11 and 12, the graphs are plotted for both latent TB and COVID-19 co-infection and active TB and COVID-19 co-infection individuals against time with varying values of $k_1$ and $k_2$. This graph shows that the different values of $k_1$ and $k_2$ in favour of $L^{TC}$ and $I^{TC}$ decrease to nearly zero. Finally, in Fig. 13, the graph is plotted for both disease recovered individuals against time and varying values of $k_1$ and $k_2$. We have found that the different values of $k_1$ and $k_2$ in favour of the number of recovered people are increasing. In Figs. 14 and 15, we plotted the graph of $I^{T}$ and $R^T$ compared to sensitive parameters infected human individuals for different values of $\rho _1$ and varying $k_1$ and $k_2$ against time. Then, the number of active TB infected human individuals decreases rapidly, and recovery from TB increases with time. At the same time, we plotted the graph of $I^C$ and $R^C$ compared to sensitive parameters in reinfected human individuals for different values of $\rho _2$ and varying $k_1$ and $k_2$ against time in Figs. 16 and 17. Then the symptomatic infection from COVID-19 and recovery from COVID-19 first increases at the initial stages and decreases with time, it gets very close to zero. Finally, the graphs are plotted to compare all compartments against time, with the same values of $k_1$ and $k_2$ $(k_1=k_2=0.95)$ in Fig. 18. Further, in Fig. 19, we obtain the simulated results with the available real data COVID-19 infected Indians in World Health Organization from $01^{st}$ June 2022 to $08^{th}$ September 2022 for 100 days as a data case and present a graphical comparison. We fixed the parameter values in these graphical results and varied the $k_1$ and $k_2$. We see that the graphs of the simulated and real data curves are very close to each other in the final stage at the order of $k_1=k_2=0.92$. Our proposed model performance is good because the number of recovered people is increasing. Hence, the fractal-fractional operator is an easy tool to understand the TB and COVID-19 co-infected model.

## Conclusions

A fractal-fractional TB and COVID-19 co-infection model is investigated in this article. Firstly, we formulated a fractal-fractional type TB and COVID-19 co-infection model to demonstrate the theoretical existence and uniqueness results under the said derivative by utilizing the fixed point approach. An examination was conducted on the criteria proposed by Ulam-Hyers stability. This paper used Lagrange polynomial interpolation to derive the numerical scheme for the TB and COVID-19 co-infection model. We can also validate the results through a numerical simulation that has been carried out for the different values for fractional order $k_1$, fractal dimensions $k_2$ and parameters. Based on the numerical simulation, we have a graphical explanation of the model and a comparison of the sensitive parameters. The numerical portion of the paper presents highly realistic graphs for various orders of $k_1$ and $k_2$. These comparative results exhibit similar patterns but with slight deviations corresponding to the specific orders of fractal-fractional derivatives. The numerical simulation shows that the fractal-fractional TB and COVID-19 model has performed very well, as the number of recovered people increases against time. To extend the research on the subject, we can use other numerical schemes and comparative analyses to the continuation of the study.

## Data Availability

All data regarding the research work is clearly mentioned in the research work.
